# Upregulation of amplified in breast cancer 1 contributes to pancreatic ductal adenocarcinoma progression and vulnerability to blockage of hedgehog activation

**DOI:** 10.7150/thno.47390

**Published:** 2021-01-01

**Authors:** Licen Li, Jiaolin Bao, Haitao Wang, Josh Haipeng Lei, Cheng Peng, Jianming Zeng, Wenhui Hao, Xu Zhang, Xiaoling Xu, Chundong Yu, Chu-Xia Deng, Qiang Chen

**Affiliations:** 1 Cancer Centre, Faculty of Health Sciences, University of Macau, Macau SAR, China.; 2 Centre for Precision Medicine Research and Training, Faculty of Health Sciences, University of Macau, Macau SAR, China.; 3 Department of Laboratory Medicine, The First Affiliated Hospital of Wenzhou Medical University, Wenzhou, Zhejiang, 325000, China.; 4 State Key Laboratory of Cellular Stress Biology, School of Life Sciences, Xiamen University, Xiamen, Fujian, 361012, China.

**Keywords:** AIB1, MafB, Hedgehog, ECM, PDAC

## Abstract

**Background:** Pancreatic ductal adenocarcinoma (PDAC) is one of the most aggressive and devastating cancers without effective treatments. Amplified in breast cancer 1 (AIB1) is a member of the steroid receptor coactivator family that mediates the transcriptional activities of nuclear receptors. While AIB1 is associated with the initiation and progression of multiple cancers, the mechanism by which AIB1 contributes to PDAC progression remains unknown. In this study, we aimed to explore the role of AIB1 in the progression of PDAC and elucidate the underlying mechanisms.

**Methods:** The clinical significance and mRNA level of AIB1 in PDAC were studied by database analysis. To demonstrate whether AIB1 mediates the malignant features of PDAC cells, namely, proliferation, migration, invasion, we performed real-time PCR and Western blot analysis, established xenograft models and used *in vivo* metastasis assay. With insights into the mechanism of AIB1, we performed RNA sequencing (Seq), ChIP-Seq, luciferase reporter assays and pull-down assays. Furthermore, we analyzed the relationship between AIB1 expression and its target expression in PDAC cells and patients and explored whether PDAC cells with high AIB1 levels are sensitive to inhibitors of its target.

**Results:** We found that AIB1 was significantly upregulated in PDAC and associated with its malignancy. Silencing AIB1 impaired hedgehog (Hh) activation by reducing the expression of smoothened (SMO), leading to cell cycle arrest and the inhibition of PDAC cell proliferation. In addition, AIB1, *via* upregulation of integrin αv (ITGAV) expression, promoted extracellular matrix (ECM) signaling, which played an important role in PDAC progression. Further studies showed that AIB1 preferably bound to AP-1 related elements and served as a coactivator for enhancing the transcriptional activity of MafB, which promoted the expression of SMO and ITGAV. PDAC cells with high AIB1 levels were sensitive to Hh signaling inhibitors, suggesting that blocking Hh activation is an effective treatment against PDAC with high AIB1 expression.

**Conclusions:** These findings reveal that AIB1 is a crucial oncogenic regulator associated with PDAC progression *via* Hh and ECM signaling and suggest potential therapeutic targets for PDAC treatment.

## Introduction

Pancreatic ductal adenocarcinoma (PDAC) is the most common malignancy of the pancreas 1. It is an aggressive disease with a poor prognosis and a five-year survival rate of approximately 9% in the United States 2. Despite enormous efforts in the development of its diagnosis and treatment, the survival rate has changed negligibly in the past few decades. By 2030, PDAC is predicted to become the second leading cause of cancer-related deaths 3. Thus, there is an urgent need to identify molecular targets for effective treatment of PDAC.

The Hedgehog (Hh) signaling pathway controls numerous processes during embryonic development. After deregulation, it contributes to tumorigenesis in a variety of human tissues 4. Hh signaling is mediated by a family of three secreted ligands, Sonic hedgehog (SHH), Indian hedgehog and Desert hedgehog. Hh signaling is activated *via* the binding of Hh ligands to the repressor Patched (PTCH). This interaction inhibits PTCH function and results in the activation of Smoothened (SMO), which in turn initiates a signaling cascade, leading to the activation of GLI transcription factors 5. It has been reported that Hh ligand expression is abnormally expressed in PDAC and is detectable throughout disease progression, even in precursor lesion-pancreatic intraepithelial neoplasia (PanIN) 6. A recent genomic study also indicated that Hh signaling is frequently increased in PDAC 7. To date, the abnormal activation of Hh signaling in cancer has been attributed to ligand-independent and ligand-dependent mechanisms 8. Activation of canonical Hh signaling through activating mutations in SMO demonstrates the essential role of this pathway in driving PDAC formation *in vivo* 9.

Integrins are heterodimeric receptors that mediate extracellular matrix (ECM) signaling through directly binding to components of ECM 10. Integrins consist of 18 α-subunits and 8 β-subunits that form at least 24 distinct integrin heterodimers 10. Dysregulated ECM signaling promotes neoplastic progression and metastasis 11. Previous studies indicate that integrin αvβ3 is expressed in approximately 58% of human PDAC cases, and it promotes tumor growth and metastasis in an anchorage-independent manner through c-Src activation 12, 13. These studies suggested that αvβ3 is associated with enhanced malignancy in PDAC.

Amplified in breast cancer 1 (AIB1), also known as steroid receptor coactivator-3 (SRC-3), is a member of the p160 SRC family which also includes SRC-1 and SRC-2. AIB1 regulates not only the transcriptional activity of nuclear receptors but also other transcription factors (TFs) such as Nrf2, E2F1, AP-1 and NF-κB 14, 15. AIB1 is an oncogene that is associated with the initiation and progression of multiple cancers, including breast cancer, prostate cancer, hepatocellular carcinoma (HCC) and cholangiocarcinoma 14-16. A previous study revealed that the expression of AIB1 is significantly higher in high grade of PanIN and PDAC 17; however, the role of AIB1 in PDAC progression remains elusive. In this study, we reveal that AIB1 is significantly upregulated in PDAC and associated with malignancy of PDAC; AIB1 interacts with MafB to promote SMO and ITGAV expression, which enhances PDAC cell proliferation and invasion *via* Hedgehog and ECM signaling.

## Materials and Methods

### Cell culture and virus infection

The following human PDAC cell lines were obtained from the American Type Culture Collection (ATCC): AsPC-1 (CRL-1682), BxPC-3 (CRL-1687), Capan-1 (HTB-79), Capan-2 (HTB-80), CFPAC-1 (CRL-1918), MIA PaCa-2 (CRL-1420), PANC-1 (CRL-1469), PANC 10.05 (CRL-2547), SU.86.86 (CRL-1837) cells. The cells were grown in 37 °C/5% CO_2_ in ATCC-recommended media. The pancreatic duct epithelial cell line HPDE6c7 was kindly provided by Dr. Ruiyu Xie (University of Macau, China). Lentivirus preparation and infection were performed as described previously 18. To establish stable AIB1 knockdown (KD) cells, PANC-1 or MIA PaCa-2 cells were infected with lentivirus-based shRNAs against AIB1 or control shRNA, and selected with 1 μg/mL puromycin for 3 weeks.

### Plasmid construction

The lentiviral shRNA plasmid pLKO.1 targeting human AIB1 (clone ID TRCN0000365196, TRCN0000370320), mouse AIB1 (TRCN0000095795), human SMO (TRCN0000378354, TRCN0000358091), human ITGAV (TRCN0000010769, TRCN0000003240), human ITGB3 (TRCN0000003237, TRCN0000003236, TRCN0000003235), human MAFB (TRCN0000017679) and a shRNA control plasmid were obtained from Sigma. The construction of pCR3.1-AIB1, pCR3.1-Flag-AIB1, and five AIB1 truncated fragments (bHLH-PAS, S/T, RID, AD1 and AD2) was performed as described previously 14. pCMV3-MafB was purchased from Sino Biological Inc. and subcloned into the pGEX-4T-1 vector. pAd MafA-I-nGFP was a gift from Douglas Melton (Addgene plasmid #19412). pCDH-EF1-Luc2-P2A-copGFP was a gift from Kazuhiro Oka (Addgene plasmid # 72485). Hh luciferase reporter plasmid (8x3'GLI-BS-delta51-LucII, RDB08061) 19 and pcDNA3.1-His-hGLI1 (RDB08063) 20 were kindly provided by RIKEN BRC. The three multimerized MAF-recognition elements (MAREs) were inserted into the pGL3-promoter vector to generate MARE-Luc reporter as described previously 21, 22. The *SMO* promoter fragment (-1433 to +175) and the *ITGAV* regulatory fragment (-14762 to -14319) were amplified and constructed into the pGL3-basic and pGL3-promoter vectors, respectively. The pRL-TK Renilla luciferase reporter construct was purchased from Promega Inc. (Madison, WI, USA).

### Cell transfection and luciferase activity assay

The cells were transfected with plasmids using Lipofectamine 3000 (Thermo Fisher Scientific). pRL-TK was cotransfected into the cells to normalize the transfection efficiency. Hh-responsive reporter assays were performed as described previously 23. NIH3T3 cells were transfected with the Hh luciferase reporter after AIB1 was silenced for 24 h. One day after transfection, the medium was replaced with the assay medium (0.5% FBS), and the indicated reagents were added to the cultures and incubated for an additional day. For measuring the luciferase activity, the cells were harvested 24 h or 48 h posttransfection and the activity was measured using a dual luciferase reporter assay system (Promega).

### Cell proliferation and viability assay

Cell proliferation and viability were analyzed by MTT and alamarBlue assay, respectively. A total of 8,000-10,000 cells were seeded in 96-well plates. For the MTT assay, MTT was added to each well and the plates were incubated for 4 h before the addition of solubilization solution (10% SDS in 0.01 M HCl). The absorbance was measured at 560 nm using a microplate reader. For the alamarBlue assay, alamarBlue™ cell viability reagent (Thermo Fisher Scientific) was added to each well 2 days after drug treatment, and the plates were incubated for 2 h and protected from direct light. The fluorescence was measured using an excitation wavelength of 560 nm and an emission wavelength of 590 nm. For the clonogenicity assay, five hundred cells were seeded in 6-well plates and cultured for 2~3 weeks. Then, colonies were stained with 0.05% crystal violet for 30 min and counted.

### Cell cycle analysis and BrdU incorporation assay

For cell cycle analysis, the cells were synchronized by serum starvation for 24 h and induced to re-enter the cell cycle by an exchange of 10% fetal bovine serum for 24 h. The synchronized cells were harvested and fixed in 75% ethanol overnight at 4 °C. Cells were treated with RNase A for 30 min at 37 °C, and then stained with propidium iodide. The cell cycle was measured by flow cytometry. For measuring BrdU incorporation, cells were cultured for 24 h after serum starvation and labeled by BrdU for 30 min. The cells were fixed and subsequently stained with PI and anti-BrdU antibody conjugated to Alexa Fluor 488 for detection by fluorescence-activated cell sorting (FACS) analysis (BD FACSCalibur).

### Cell migration and invasion

Cell migration and invasion assays were performed using CytoSelect™ 24-well Cell Migration Assay kit (Cell Biolabs Inc.) or BD BioCoat™ Tumor Invasion System with Falcon FluoroBlok 24-Multiwell Insert Plate (BD Biosciences), respectively. Briefly, 1-2 ×10^5^ PANC-1 or MIA PaCa-2 cells were seeded into the upper chambers in serum-free DMEM, whereas the lower chambers were loaded with DMEM containing 10% FBS. Then, the migrating or invasive cells were measured according to the manufacturer's instructions. Cell migration was also measured by xCELLigence real time cell analyzer (RTCA) System with CIM-Plate according to the manufacturer's instructions (Roche).

### RNA-Seq and real-time PCR

Total RNA was isolated with TRIzol reagent (Thermo Fisher Scientific) according to the manufacturer's instructions. For RNA-Seq, RNA was processed using the RNeasy mini kit (QIAGEN), and RNA concentration and integrity were measured using an Agilent 2100 Bioanalyzer (Agilent Technologies). cDNA libraries were prepared from RNA (RIN values >8.0), using NEBNEXT® Ultra™ RNA Library Prep Kit for Illumina (New England Biolabs) according to the manufacturer's instructions, and libraries' quality was verified with Agilent 2100 Bioanalyzer. Nine libraries were generated with PNAC-1 expressing shCtrl (3), shAIB1-1 (3) or shAIB1-2 (3), which were paired-end sequenced by HiSeq 2500 (Illumina) in Genomics, Bioinformatics and Single Cell Analysis Core, Faculty of Health Science, University of Macau. Reverse transcription was performed using a QuantiTect Reverse Transcription Kit (QIAGEN). Real-time PCR was performed using FastStart Universal SYBR Green Master (Roche) on a QuantStudio™ 7 Flex Real-Time PCR System (Thermo Fisher Scientific). The relative quantification was determined by normalizing the values to the amount of 18S. The primers used for real-time PCR are listed in Table S1.

### Pathway enrichment analysis and gene set enrichment analysis (GSEA)

The quality of the sequencing data was analyzed using FastQC (version 0.11.5), and raw reads with low quality were removed using Trim Galore (version 0.4.4) prior to analysis of the data. All the trimmed reads were mapped to the reference genome (hg38, GRCh38) by STAR software (version 020201), and the mapped counts were extracted using the feature count from the Subread package (version 1.5.3). Subsequently, read count data containing 26,643 transcripts quantified with raw reads were preprocessed by filtering out genes with a zero read count across different samples, and 19,020 genes remained after filtering. The read count data was normalized by DESeq2, which can be used for downstream differential expression analysis. Differentially expressed genes (DEGs) in the AIB1-KD cells compared to the control cells were filtered by log2-fold change > 1 or < -1. A *P* value < 0.05 was considered to indicate a significant difference. GSEA was performed using the R package clusterProfiler 24 with the normalized enrichment score calculated by creating 1,000 permutations of the ES and scaling the observed ES by the mean score of the permutations. The gene sets implemented were derived from the Cancer Hallmark Gene, Kyoto Encyclopedia of Genes and Genomes (KEGG) pathway and Gene Ontology (GO) database, as collected in the Molecular Signatures Database (MSigDB; version 6.2). GSEA was applied to the selected gene sets to determine gene enrichment in each dataset. All data processing was completed in a shell HPC command line environment.

### Chromatin immunoprecipitation (ChIP) and ChIP-Seq analysis

PANC-1 cells were cross-linked by 1% formaldehyde at room temperature for 10 min. The cross-linking reaction was stopped by adding glycine to a final concentration of 0.125 M, and then the cells were washed with cold PBS and scraped from dishes. After pelleting the cells, the samples were resuspended by RIPA buffer and processed with Bioruptor Twin (Diagenode) in a circulating water bath according to the manufacturer's instructions. Then, the supernatants were collected for immuneprecipitation. After immunoprecipitation with antibodies or control IgG, the samples were purified by a QIAquick PCR Purification Kit (Qiagen) and measured by real-time PCR. The primers used for real-time PCR are listed in Table S1. For ChIP-Seq, the samples were sequenced by Novogene Co., Ltd. The quality of the sequencing data was analyzed by FastQC (version 0.11.5), and raw reads with low quality were removed using Trim Galore (version 0.4.4) prior to analysis of the data. All the trimmed reads were mapped to reference genome (hg38, GRCh38) using the bowtie2 tool with default settings, and SAM format files were processed using SAMtools 25 and sambamba 26. Then, the peaks were called by MACS2 27 with -f BAM -g hs -q 0.01 for AIB1 IP compared with the corresponding input. The BAM files and peak files contained the input of ChIPQC to generate basic reports, including the percentage of reads within peaks.

### Focal adhesion assay

Vitronectin (0.5 μg/cm^2^) was used to coat culture vessels for the focal adhesion assay. For focal adhesion formation, cells were seeded in the 24-well plates for 3 h, and then fixed and stained with Focal Adhesion Staining Kit (FAK100, Millipore). The number of focal adhesion was counted by ImageJ software according to a previous study 28. For focal adhesion kinase (FAK) phosphorylation, the cells were starved in medium with 0.5% FBS overnight, seeded in 6-well plates and harvested at different time points.

### Western blotting

Equal amounts of protein lysates were separated by SDS-PAGE and transferred onto PVDF membranes. The primary antibodies used for Western blotting are listed in Table S2. After extensive washing, the membranes were then incubated with horseradish peroxidase conjugated secondary antibody (Cell Signaling Technology) and visualized by chemiluminescence (Immobilon ECL Ultra Western HRP Substrate, Millipore).

### Co-immunoprecipitation (IP) and GST pull-down assay

For co-IP, the plasmids expressing Flag-AIB1 or MafB were cotransfected into 293T cells, and the cells were lysed 24 h after transfection. The cell lysates were immunoprecipitated with the corresponding antibodies or control IgG. After washing, the precipitates were analyzed by western blotting. For the GST pull-down assay, the GST-MafB proteins or GST were incubated with glutathione Sepharose 4B beads (GE Healthcare), followed by the addition of the lysates of cells overexpressing Flag-AIB1 or the Flag-AD2 domain. After incubation, the beads were washed and analyzed by western blotting.

### Animal study

To generate Luc-GFP cells, PANC-1 cells were infected with lentivirus carrying luciferase/GFP cDNA, and GFP positive cells were sorted by FACS (BD FACSAria™ III). Six to eight-week-old male severe combined immunodeficient (SCID) mice were obtained from the Animal Facility of Faculty of Health Sciences, University of Macau. Xenograft assays were performed in two batches. First, 2×10^6^ shAIB1-2 and shCtrl PANC-1 cells were subcutaneously (SC) inoculated into the dorsal flanks of the SCID mice, and then, after the establishment of tumors, tumor size and weight were measured. In the second batch, 2×10^6^ shAIB1-2 and shCtrl Luc-GFP cells were SC injected into the mice. After inoculation for 50 days, the luciferase activity in the tumors was detected after the mice were intraperitoneally injected with D-luciferin (150 mg/kg body weight, Promega) for 10-15 min. For the metastasis assay, 0.5×10^6^ shAIB1-2 and shCtrl Luc-GFP cells were intravenously injected into the mice through tail vein. One month after injection, the lungs were removed and observed under a fluorescence stereomicroscope. For drug treatment, MIA PaCA-2 or AsPC-1 cells were implanted into SCID mice, which were randomly assigned to two groups when the tumor diameter reached approximately 0.5 cm. The mice were treated with either vehicle (control) or sonidegib (20 mg/kg). All experimental procedures involving animals were approved by the University of Macau Animal Ethics Committee under the protocol (UMAEC-050-2015).

### Immunohistochemistry (IHC)

Deparaffinized tumor sections were cooked with Retriever (Electronic Microscopy Science) in buffer A (citrate buffer [pH 5.0]), followed by incubation with the primary antibody. Then, the sections were developed using a Histostain-Plus IHC Kit (Thermo Fisher Scientific) and counterstained with Hematoxylin.

### Statistical analysis

All values are expressed as the mean ± SEM of individual samples. The data are representative of at least two independent experiments. The samples were analyzed by unpaired two-tailed *t*-test or one/two-way ANOVA. Kaplan-Meier curve was generated by using Kaplan-Meier Plotter with the best expression cut-off (http://kmplot.com/analysis/). Values of *P* less than 0.05 were considered statistically significant. Statistical analysis was performed using GraphPad Prism 7.

## Results

### Upregulation of AIB1 facilitates the proliferation and metastasis of PDAC cells

**Figure 1:**
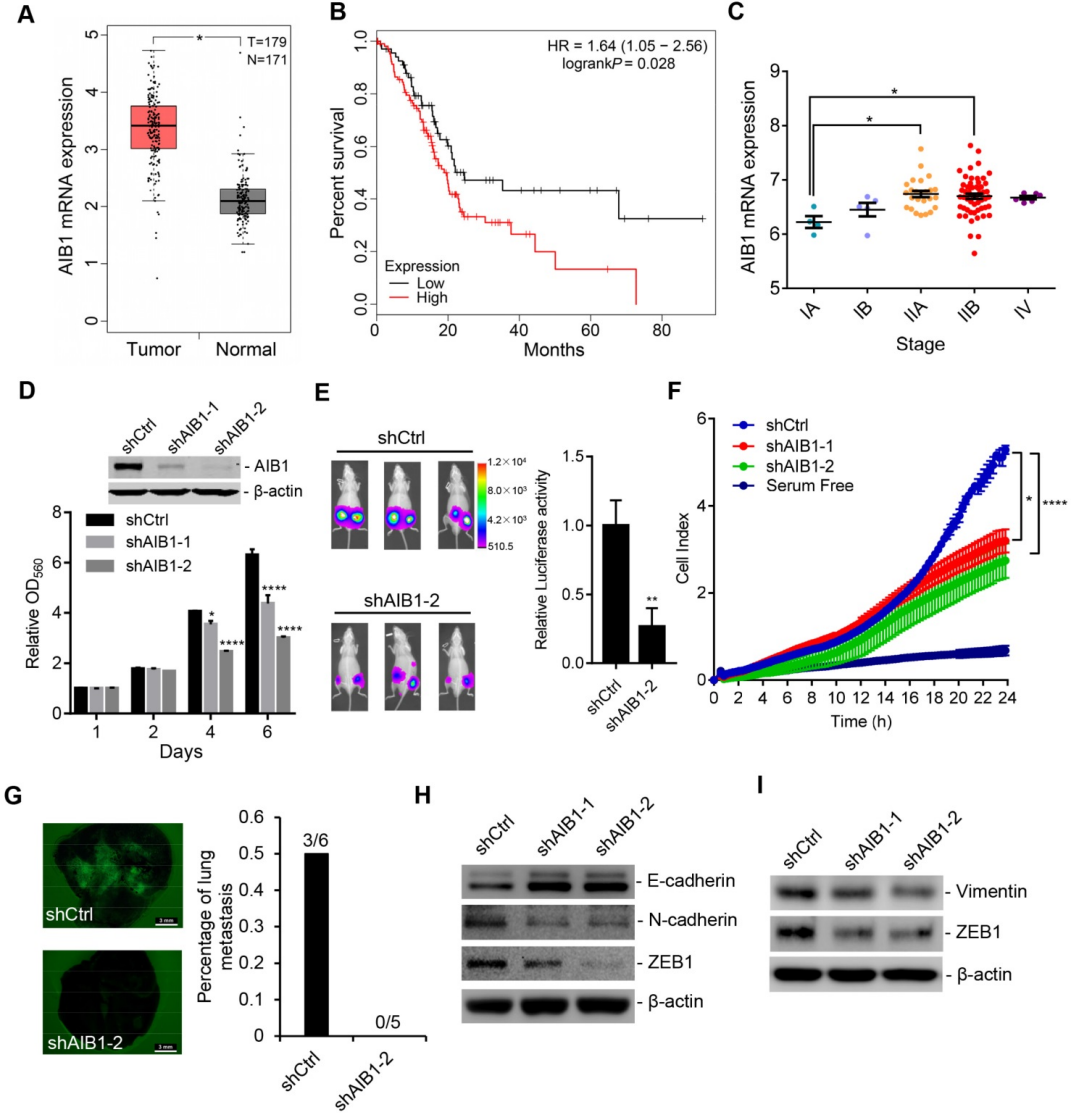
** AIB1 is upregulated in PDAC and promotes PDAC progression.** (A) AIB1 mRNA levels in normal pancreatic (N) and PDAC (T) tissues. (B) Kaplan-Meier curve showing the overall survival of PDAC patients with low (n=71) or high AIB1 (n=106) levels. The log rank (Mantel-Cox) *P* value is indicated. (C) AIB1 mRNA levels in different stages of PDAC according to the American Joint Committee on Cancer TNM system. (D) The effect of knocking down AIB1 on the proliferation of PANC-1 cells. Upper panel: Western blot showing AIB1; lower panel: quantification of cell proliferation as assessed by MTT assay. (E) Knocking down AIB1 with shAIB1-2 decreases tumors consisting of PANC-1 cells *in vivo*. Left panel: representative images of the mice inoculated with Luc-GFP cells; right panel: quantitation of the luciferase activity of tumors, n=6 per group. (F) The effect of AIB1 on the migration of PANC-1 cells as measured by xCELLigence RTCA system. (G) Lung metastasis of PANC-1 cells *in vivo*. Scale bar, 3 mm. Left panel: representative image of lungs; right panel: quantitation of lung metastasis. (H, I) knocking down AIB1 impaired the EMT process and inhibited ZEB1 expression in PANC-1 (H) and MIA PaCa-2 (I) cells. Data are presented as the means ± SEM. *P < 0.05, **** P < 0.0001 as determined by unpaired two-tailed *t*-test (A, E), one-way (C, F) or two-way (D) ANOVA.

To investigate the clinical significance of AIB1, we analyzed AIB1 mRNA levels in normal pancreatic and adenocarcinoma tissues that were extracted from The Cancer Genome Atlas (TCGA) and Genotype-Tissue Expression (GTEx) database by GEPIA 29. We found that AIB1 was significantly increased in tumor tissues compared with normal tissues; and higher AIB1 expression was associated with worse prognosis (Figure 1A-B). Considering a recent study 30, we obtained AIB1 mRNA levels at different TNM stages by cBioPortal 31 and found that the expression of AIB1 was related to PDAC progression (Figure 1C). Furthermore, we measured AIB1 levels in different PDAC cell lines and immortalized pancreatic duct epithelial cells (HPDE6c7). The results showed that AIB1 expression was upregulated in most PDAC cell lines, except in AsPC-1 and Capan-2 cells, compared to that in the HPDE6c7 cells (Figure S1A). These data suggested that AIB1 may play a role in the development of PDAC.

To investigate whether AIB1 has effects on PDAC progression, we chose two PDAC cell lines (PANC-1 and MIA PaCa-2) with high AIB1 abundance, and AIB1 expression was stably silenced in these cells by infection with two different lentiviral shRNAs against AIB1 (shAIB1-1 and shAIB1-2). The downregulation of AIB1 in shAIB1 cells was confirmed by Western blot analysis after comparison to the level in the control cells infected with control shRNA (shCtrl) lentivirus (Figure 1D and Figure S1B). Although there was no significant difference in proliferation between shCtrl cells and shAIB1 cells in the first two days, we found that knockdown of AIB1 obviously suppressed cell proliferation after 4 and 5 days in culture and blocked colony formation (Figure 1D and Figure S1B-D). In addition, the results indicated that knocking down AIB1 impaired the entry of cells into S phase (Figure S1E), and induced an accumulation of cells with a 4N DNA content (Figure S1F), suggesting G2/M arrest or failure of cytokinesis. Next, to verify the role of AIB1 in PDAC growth *in vivo*, we compared the growth of shAIB1 and shCtrl PANC-1 xenograft tumors in the SCID mice. PANC-1 cells with shAIB1-2 formed smaller tumors compared to those formed with shCtrl cells, as indicated by tumor volume and weight (Figure S1G). In addition, we generated luciferase-GFP labeled PANC-1 cells (Luc-GFP cells) with shCtrl or shAIB1-2. The results showed that luciferase activity in the AIB1-KD cells was dramatically decreased compared to that in the shCtrl cells (Figure 1E). Consistently, PCNA, as a proliferative marker, was significantly downregulated in AIB1-KD tumors compared to its expression in control tumors (Figure S1H). These data demonstrate that AIB1 is required for the growth of PDAC cells both *in vitro* and *in vivo*.

To further address whether AIB1 participates in the migration and invasion of PDAC cells, we conducted real-time monitoring of shCtrl and shAIB1 PANC-1 cell migration using a RTCA system. The results showed that knocking down AIB1 significantly blocked the migration of PANC-1 cells (Figure 1F). Then, we performed Transwell assays to determine the effect of AIB1 on the invasiveness of PANC-1 and MIA PaCa-2 cells. The data corroborated that the downregulation of AIB1 reduced the migration and invasion ability of these PDAC cell lines (Figure S2A-D). Furthermore, we investigated the effect of AIB1 on the metastasis of PDAC cells *in vivo* by inoculating Luc-GFP cells into SCID mice by tail vein injection. Although we did not capture the luciferase signal *in vivo* due to detector insensitivity, we found that three of six lungs in the control group showed GFP signal, and none of five lungs in the AIB1-KD group had GFP signal (Figure 1G). To verify that AIB1 has a crucial role in PDAC metastasis, we also examined whether AIB1 affects the metastasis of PDAC cells to the lungs in an early stage. The results showed that knocking down AIB1 significantly impaired the colonization of PDAC cells within 36 h after inoculation (Figure S3). These data indicate that AIB1 plays an important role in promoting PDAC metastasis.

The activation of the epithelial-mesenchymal transition (EMT) is considered a driver of tumor progression from initiation to metastasis 32. Cadherin switching (the downregulation of epithelial E-cadherin and upregulation of mesenchymal N-cadherin expression) is a major hallmark of the EMT 32. Our data showed that cadherin switching was impaired in the AIB1-KD PANC-1 cells, which indicates that knocking down AIB1 can block the activation of the EMT (Figure 1H). Similarly, we found that knocking down AIB1 inhibited the expression of vimentin, a mesenchymal marker, in MIA PaCa-2 cells lacking cadherin (Figure 1I) 33. The transcriptional repressor zinc‐finger E‐box binding homeobox 1 (ZEB1) is a crucial activator of the EMT in various human tumors and has recently been shown to promote metastasis in pancreatic cancer 34, 35. We found that knocking down AIB1 repressed the expression of ZEB1 in PANC-1 and MIA PaCa-2 cells at both the protein and mRNA levels (Figure 1H-I and Figure S2E-F), suggesting that AIB1 regulates the EMT by mediating the expression of ZEB1.

Taken together, our results demonstrate that AIB1 is required for the proliferation and invasion of PDAC cells and plays an important role in promoting PDAC progression.

### AIB1 promotes cell proliferation by regulating SMO expression and hedgehog signaling activation

**Figure 2:**
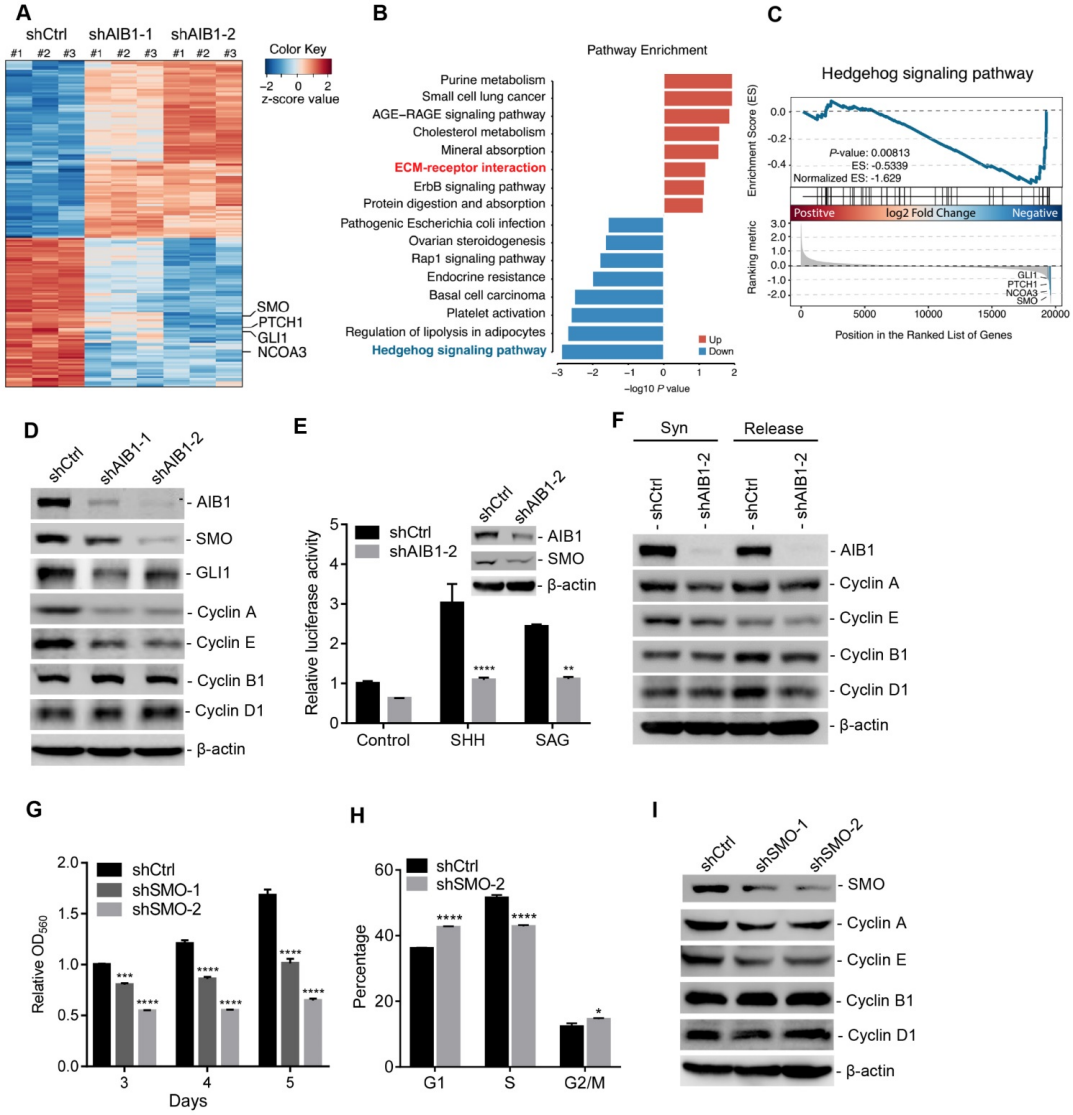
** Downregulation of AIB1 impairs the proliferation of PDAC cells by blocking Hh activation.** (A) Heatmap of DEGs in AIB1-KD PANC-1 cells compared to control cells. (B) Top pathways of the upregulated-DEGs and the downregulated-DEGs in AIB1-KD PANC-1 cells versus control cells as determined by GO analysis. (C) GSEA plot of enrichment in “Hedgehog signaling pathway” gene set, significantly downregulated in AIB1 KD PANC-1 cells with shAIB1-2. (D) The effect of AIB1 on SMO, GLI1 and cell cycle regulatory protein levels in PANC-1 cells as analyzed by Western blotting. (E) Knocking down AIB1 impairs the Hh activation induced by SHH (1 μg/mL) or SAG (100 nM). AIB1 and SMO levels are shown in the inset figure. (F) Knocking down AIB1 caused cell cycle arrest by reducing the expression of cyclin A and E. Syn, synchronized by double-thymidine block; Release, 6 h after thymidine was removed. (G) The effect of SMO on proliferation of PANC-1 cells, according to MTT assay. (H) Cell cycle distribution of the shCtrl and shSMO PANC-1 cells. (I) The effect of SMO on cell cycle regulatory protein levels in PANC-1 cells. Data are presented as the means ± SEM. *P* < 0.05, **P < 0.01, ***P < 0.001, ****P < 0.0001 as determined by two-way ANOVA (F, G, and H).

To explore the relevant molecular mechanism by which AIB1 promotes PDAC progression, we performed transcriptome analysis in stable shCtrl- and shAIB1-expressing PANC-1 cells by RNA-Seq. As shown in Figure 2A, knocking down AIB1 caused many changes in gene expression and the two shAIB1 groups showed the similar pattern compared to shCtrl group. Considering the GSEA, we found that knocking down AIB1 dramatically impaired the G2/M transition pathway and promoted the cell cycle arrest regulatory pathway (Figure S4A), confirming that the downregulation of AIB1 causes cell cycle arrest (Figure S1F). Our previous studies indicated that AIB1 drives tumor progression by enhancing AKT and NF-κB activation 14, 15. However, the RNA-Seq analysis indicated that knocking down AIB1 had no effect on these two pathways in PANC-1 cells (Figure S4A). Consistently, the results also showed that knocking down AIB1 had no effect on AKT phosphorylation in PANC-1 or MIA PaCa-2 cells (Figure S4B), suggesting that AIB1 can promote PDAC progression independent of AKT activation.

Of note, pathway enrichment analysis revealed that knocking down AIB1 significantly suppressed Hh signaling pathway, and GSEA also showed this pathway was downregulated in shAIB1 cells compared to shCtrl cells (Figure 2B-C). Then, we found that SMO, a key upstream component of the Hh signaling pathway, was dramatically decreased in AIB1-KD cells, which was confirmed by real-time PCR (Figure 2A and Figure S4C-D). In addition, downstream targets of Hh signaling pathway including *GLI1* and *PTCH1* were also significantly reduced in AIB1-KD cells (Figure 2A and Figure S4E-F), indicating that AIB1 can regulate Hh pathway activation. To determine whether AIB1 directly affects Hh activation by regulating the expression of SMO, we first performed transient silencing of AIB1 in PANC-1 and MIA PaCa-2 cells. The results revealed that transient knockdown of AIB1 inhibited SMO and its target GLI1 expression indicating that AIB1 can directly regulate the expression of SMO (Figure 2D and Figure S4G). Then, we conducted Hh luciferase reporter assay to examine the effect of AIB1 on Hh activation induced by Hh agonists such as SHH and SAG, as suggested by previous studies 19, 23. The results showed that knocking down AIB1 significantly blocked Hh activation under SHH or SAG treatment, but had no effect on the activation induced by GLI1 (Figure 2E and Figure S4H). This demonstrates that AIB1 mediates Hh signaling activation by regulating SMO expression not by serving as a coactivator for transcription factor GLI1.

It has been reported that Hh signaling can regulate cell proliferation *via* promoting the expression of genes involved in the progression of cell cycle 5. To address the underlying mechanism of cell cycle arrest caused by knocking down AIB1 in PDAC cells, we examined whether AIB1 affected the expression of key cell cycle proteins. The results showed that knocking down AIB1 reduced the levels of Cyclin A and E, but had no effect on the levels of Cyclin B1 or D1 in PANC-1 cells (Figure 2D), which was further validated in MIA PaCa-2 cells and xenograft tumors (Figure S4G and Figure S5). To confirm that AIB1 directly affects cyclin expression, and does not inhibit their expression through induced cell cycle arrest, we synchronized the cells by double-thymidine block and then checked the levels of cyclins after synchronization or release for 6 h. The results showed that Cyclin A and E in AIB1-KD cells decreased even after the cells were synchronized (Figure 2F). These results indicate that AIB1 promotes the proliferation of PDAC cells through regulating cell cycle proteins. To explore whether AIB1 regulates PDAC cell proliferation through Hh signaling, we performed transient silencing of SMO in PANC-1 and MIA PaCa-2 cells. The results presented that knocking down SMO also blocked cell proliferation and caused cell cycle arrest by inhibiting the expression of Cyclin A and E (Figure 2G-I and Figure S4I-J), which was consistent with the findings from AIB1-KD cells. Therefore, we conclude that AIB1 plays a crucial role in the control of Hh signaling by regulating SMO expression, which promotes the proliferation of PDAC cells.

### AIB1 enhances cell migration by promoting ECM signaling activation

Previous study indicates that enhanced Hh signaling promotes cancer cell migration, invasion and metastasis 36, 37, which suggests that AIB1 may also affect PDAC cell migration *via* regulating Hh signaling. However, we found that knocking down SMO had no effect on the migration of PANC-1 cells (Figure S6), indicating that AIB1 promotes PDAC cell migration independent of Hh signaling. When performing pathway enrichment analysis, the ECM-receptor interaction pathway, an upregulated pathway in AIB1-KD cells, caught our attention (Figure 2B). ECM signaling is mediated by integrins directly interacting with components of the ECM and plays an important role in promoting PDAC progression 38. We found that 16 genes were enriched in the ECM-receptor interaction pathway, and were mainly associated with the extracellular matrix, such as collagen and laminin (Figure S7A). However, the expression of these genes was inconsistent in the two AIB1 shRNA groups, suggesting that changes in the expression of these genes may be the secondary events caused by stable AIB1 silencing. To verify this hypothesis, we measured the expression of these genes in PANC-1 cells after infection with lentiviral shRNA for two days. As shown in Figure S7B, acute knockdown of AIB1 had no effect on the expression of these genes, except for certain genes showing inconsistent patterns in the two different AIB1 shRNA groups. This indicates that the upregulated ECM-receptor interaction pathway in AIB1-KD cells is a secondary event caused by the stable knockdown of AIB1.

**Figure 3:**
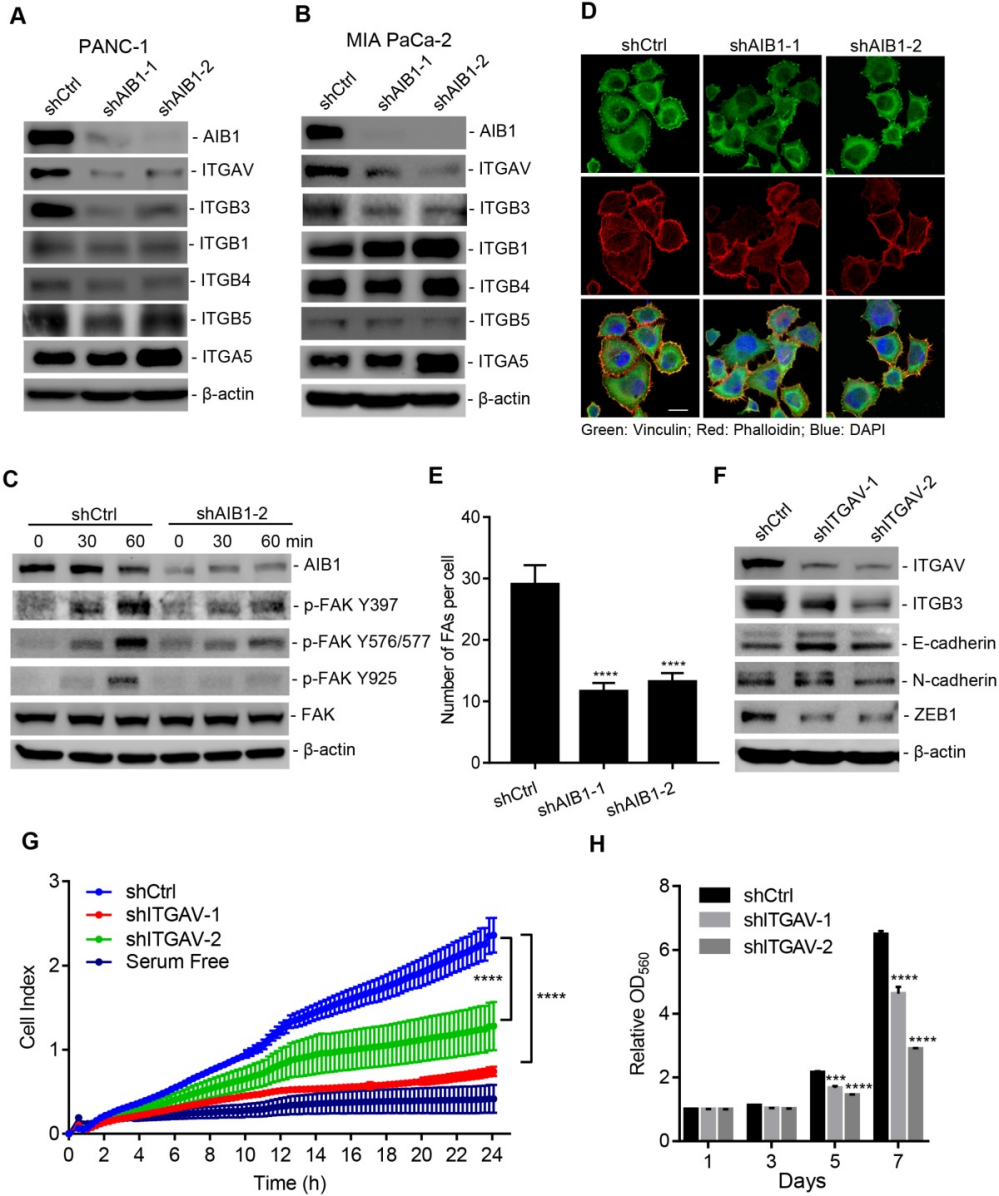
** The downregulation of AIB1 suppresses ECM signaling by decreasing ITGAV level.** (A, B) The effect of AIB1 on integrin levels in PANC-1 (A) and MIA PaCa-2 (B) cells. (C) Knocking down AIB1 impairs vitronectin-induced ECM signaling activation, as determined by measuring phosphorylation of FAK. (D, E) Knocking down AIB1 blocks focal adhesion (FA) formation on vitronectin-coated slides. (D) Representative images of FAs by immunostaining, Scale bar, 20 µm; (E) Quantification of the FAs in each cell, based on at least 10 fields counted for each group. (F, G) Knockdown of ITGAV induced cadherin switching (F) and migration (G) of PANC-1 cells. (H) Knockdown of ITGAV decreased the proliferation of PANC-1 cells. Data are presented as the means ± SEM. ***P < 0.001, ****P < 0.0001 as indicated by one (E and G) or two-way (H) ANOVA.

To understand how loss of AIB1 causes upregulation of ECM-related genes (Figure S7A), we further analyzed the expression of integrins, which are receptors that facilitate ECM signaling. Surprisingly, we found that integrin αv (ITGAV), which forms a heterodimer with integrin β3 (ITGB3), integrin αvβ3, was significantly downregulated in PANC-1 and MIA PaCa-2 cells after AIB1 was silenced by two different shRNAs (Figure 3A-B and Figure S8A-B). Interestingly, although knocking down AIB1 had no effect on ITGB3 mRNA expression, it significantly inhibited ITGB3 protein levels in both cell lines (Figure 3A-B and Figure S8A-B). In addition, ITGB5 expression was significantly decreased in AIB1-KD PANC-1 cells, but not in MIA PaCa-2 cells. These data imply that AIB1 can mainly regulate the expression of ITGAV to affect ECM signaling.

It has been reported that integrin α_v_β_3_ is upregulated in PDAC and promotes its metastasis 12, 13, which suggests that AIB1 may enhance the migration of PDAC cells by mediating ECM signaling. Therefore, we examined whether AIB1 is involved in ECM signaling activation. Integrin bind to the ECM and their subsequent clustering induces the activation of downstream signaling cascades, leading to the phosphorylation of FAK. Integrin α_v_β_3_ acts as a vitronectin receptor because vitronectin is the major ECM ligand for α_v_β_3_ 39. Therefore, we incubated shAIB1 and shCtrl PANC-1 cells *in vitro*nectin-coated dishes and assessed adhesion-induced FAK phosphorylation. The results revealed that there was no difference in total FAK levels between shAIB1 and shCtrl PANC-1 cells, whereas the phosphorylation of FAK at different sites (Y397, Y576/577, and Y925) in shAIB1 cells was dramatically inhibited at the indicated time points (Figure 3C). FAK mediates focal adhesion formation through tyrosine phosphorylation during cell adhesion. As expected, knocking down AIB1 impaired focal adhesion formation on vitronectin-coated slides compared to the effect of control cells (Figure 3D-E), which verified the crucial role of AIB1 in the control of ECM signaling. Next, to examine whether ECM signaling is involved in the progression of PDAC, we performed transient silencing of ITGAV in PANC-1 cells. The results indicated that knocking down ITGAV inhibited cadherin switching and decreased the expression of ZEB1 (Figure 3F), which was consistent with shown in AIB1-KD cell experiments (Figure 1H). Since the EMT is considered the crucial step in tumor metastasis, blocking the EMT might lead to impairment of the migrative and clonogenic capacity following knocking down ITGAV. We found that knocking down ITGAV significantly blocked the migration and proliferation of PANC-1 and MIA PaCa-2 cells (Figure 3G-H and Figure S9). Interestingly, the results showed that knocking down ITGAV caused a reduction in ITGB3 at the protein level but not at the mRNA level (Figure 3F and Figure S8C). Although knocking down ITGB3 impaired cadherin switching, it had no effect on ITGAV expression (Figure S8C-D). Therefore, these data indicate that AIB1 positively regulates the expression of ITGAV, thereby affecting the abundance of ITGB3, and enhancing ECM signaling.

### AIB1 cooperates with the transcriptional factor MafB to promote the expression of SMO and ITGAV

Our results demonstrated that AIB1 can enhance Hh and ECM signaling by regulating SMO and ITGAV expression, respectively. To confirm the effect of AIB1 on their expression in PDAC cells, AIB1 was overexpressed in Capan-2 cells with low levels of AIB1 (Figure S10A). The results showed that AIB1 increased the levels of SMO and ITGAV in a dose-dependent manner. In addition, we found that knocking down AIB1 significantly decreased the levels of SMO and ITGAV in xenograft tumors (Figure S10B). In addition, clinical data analysis also showed a positive correlation of AIB1 levels with SMO and ITGAV levels, respectively (Figure S10C). Therefore, these data indicate that AIB1 regulates the expression of SMO and ITGAV both *in vitro* and *in vivo*.

**Figure 4:**
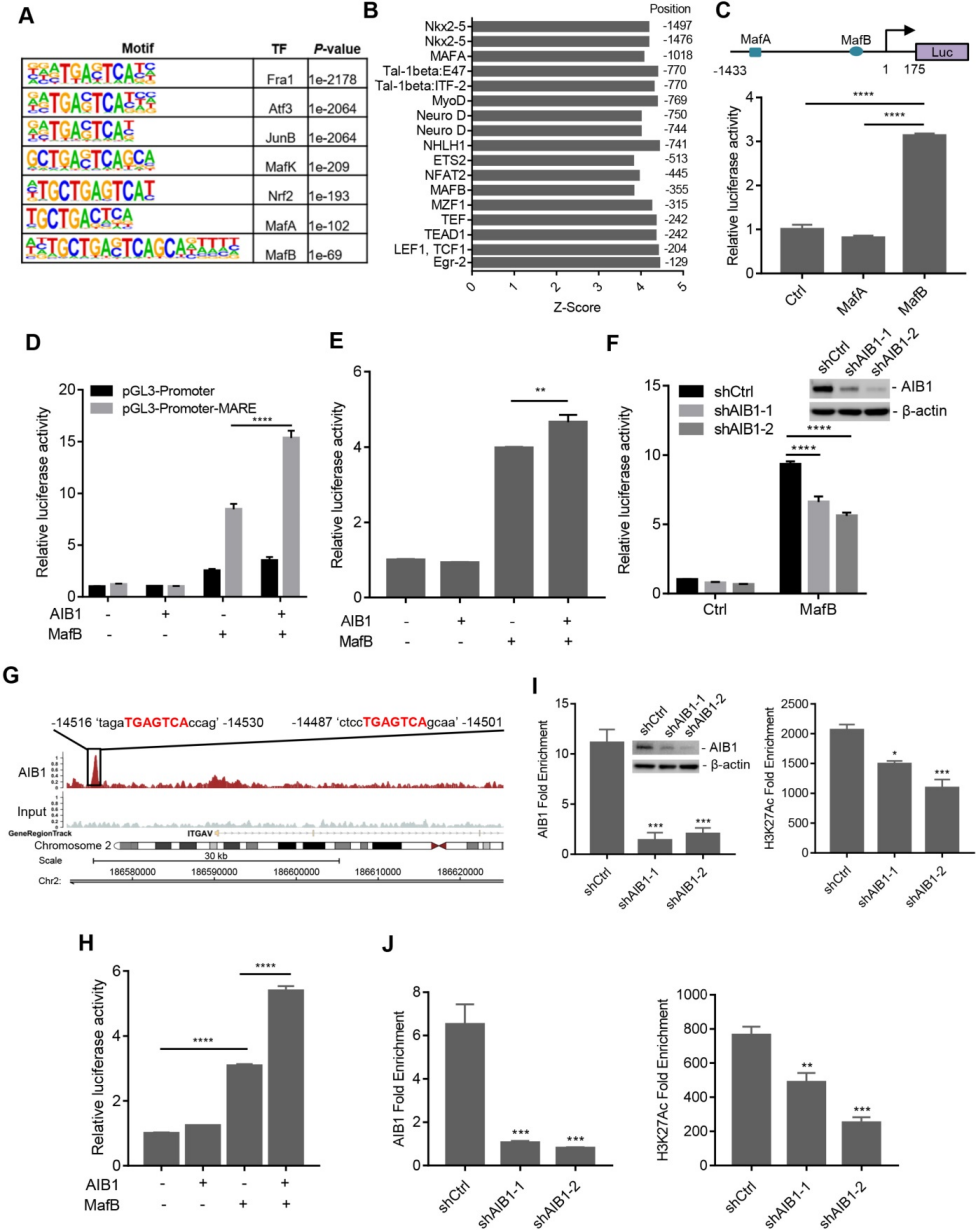
** AIB1 interacts with MafB and promotes the expression of SMO and ITGAV by mediating MafB transcriptional activity.** (A) The significant motifs identified from AIB1 ChIP-Seq. (B) In silico analysis of the *SMO* promoter. (C) The luciferase activity of the *SMO* promoter reporter (upper panel) following ectopic expression of MafA or MafB. (D) AIB1 enhances MafB‐mediated MARE‐promoter activity in PANC-1 cells, and cells transfected with pGL3-Promoter are the controls. (E) AIB increases the activity of the *SMO* promoter in the presence of MafB. (F) Knocking down AIB1 impairs MafB-mediated* SMO* promoter activity. (G) The binding peak of AIB1 at the regulatory region of *ITGAV*, which contains two MAREs, in red. (H) AIB1 enhances MafB‐mediated activity of the *ITGAV* regulatory region in PANC-1 cells. (I) AIB1 binds to the promoter of *SMO* and regulates H3H27Ac levels at the promoter. (J) AIB1 is recruited to the regulatory region of *ITGAV* and increases H3H27Ac levels. Data are presented as the means ± SEM. *P < 0.05, **P < 0.01, ***P < 0.001, ****P < 0.0001 as indicated by one (C, E, H, I and J) or two-way (D and F) ANOVA.

AIB1, a transcriptional coactivator, is recruited to DNA regulatory regions by nuclear receptors or other transcription factors to enhance regulatory activity. Therefore, AIB1 may engage with certain transcription factors to promote the expression of target genes. To investigate this hypothesis, we performed ChIP-Seq assay to identify AIB1-associated DNA binding motifs. Through bioinformatics analysis, we found that AIB1 mainly binds to the consensus sequence for the AP-1 transcription factor superfamily, including Fos, Jun, ATF, and Maf subfamilies (Figure 4A). AIB1 was also recruited to the Nrf2 related element containing a similar AP-1 like binding motif, a finding consistent with that of our previous study 14. Therefore, AIB1 may promote the expression of SMO and ITGAV *via* these associated transcription factors.

To verify the specific transcription factor involved in AIB1-mediated target gene expression, we first predicted the TF motifs at the putative promoter of human *SMO* using online resource (http://motifmap.ics.uci.edu) 40, 41. We identified two Maf-binding sites for MafA and MafB, which are -1018 and -355 upstream of the *SMO* gene, respectively (Figure 4B). To validate whether AIB1 regulates the promoting activity of *SMO via* Maf, we generated a luciferase reporter with the *SMO* promoter encompassing the two Maf-binding motifs (Figure 4C). We performed luciferase reporter assay following cotransfection of the reporter with MafA or MafB into PANC-1 cells and observed that MafB significantly enhanced luciferase activity compared to that of control or MafA group (Figure 4C). These results suggest that AIB1 may cooperate with MafB to regulate the expression of its target genes. Then, we cloned MARE into a pGL3-promoter vector and examined the effect of AIB1 on MafB-mediated transcriptional activity. As shown in Figure 4D, a significant increase in the luciferase activity was observed in the reporter carrying MARE with the combination of AIB1 and MafB compared to the effect of MafB alone, which indicates that AIB1 can promote the transcriptional activity of MafB. Furthermore, we investigated whether AIB1 cooperated with MafB to enhance *SMO* promoter activity. The results revealed that although AIB1 had no effect on *SMO* promoter activity in the absence of MafB, AIB1 increased *SMO* promoter activity in the presence of MafB (Figure 4E). In contrast, knocking down AIB1 suppressed MafB-mediated *SMO* promoter activity in PANC-1 and MIA PaCa- cells (Figure 4F and Figure S11). These results demonstrate that AIB1 directly regulates the expression of SMO which is dependent on MafB.

Next, to determine whether AIB1 regulates the expression of ITGAV by mediating MafB transcriptional activity, we analyzed the ChIP-Seq data to assess AIB1 genomic binding. An AIB1 binding peak localized at the -14.5 kb position from the upstream region of the *ITGAV* gene was observed, and it contained two Maf-binding motifs according to the analysis by Genomatix software (Figure 4G). This finding suggests that AIB1 may also cooperate with MafB to promote ITGAV expression *via* this region. Therefore, we generated the luciferase reporter by inserting this region into a pGL3-Promoter vector and examined whether AIB1 regulated the luciferase activity through this region. As shown in Figure 4H, AIB1 without MafB had no effect on the luciferase activity compared to that of the control group, but AIB1 dramatically promoted the luciferase activity induced by MafB, indicating that AIB1-regulated expression of ITGAV is dependent on MafB.

AIB1 contains an intrinsic histone acetyltransferase (HAT) activity and recruits other coactivators such as CBP/p300, to induce the acetylation of histones, which makes downstream DNA more accessible to transcription 16. We found that knocking down AIB1 significantly reduced the binding of AIB1 to the *SMO* promoter and the level of H3K27Ac, a histone mark indicative of an active promoter or enhancer (Figure 4I). Similarly, silencingAIB1 decreased the levels of AIB1 and H3K27Ac in the regulatory region of *ITGAV* (Figure 4J). Taken together, these data indicate that AIB1 facilitates MafB induction of SMO and ITGAV expression in PDAC cells.

**Figure 5:**
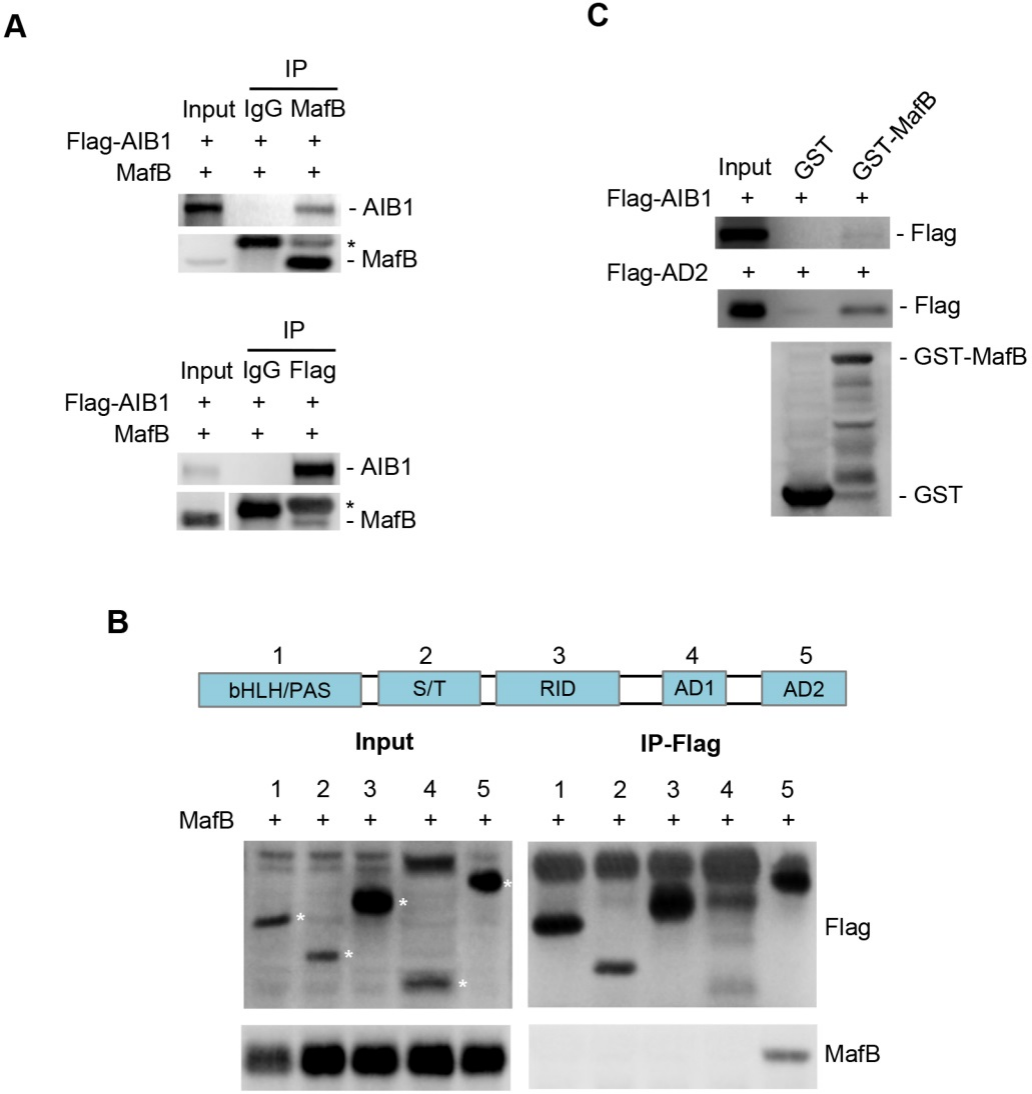
** AIB1 interacts with MafB through the AD2 domain of AIB1.** (A) Co-IP analysis of the interaction of AIB1 and MafB in 293T cells transfected with MafB and Flag-AIB1 expressing vector. Asterisk (*) indicates the IgG band. (B) AIB1 interacts with MafB through its AD2 domain. (C) The interaction of AIB1 with MafB was confirmed by GST pull‐down assay.

AIB1 contains several functional regions, such as basic helix-loop-helix region with a PAS (Per/ARNT/Sim) motif (bHLH/PAS), serine/threonine-rich region (S/T), receptor-interaction domain (RID), and the C-terminal region containing two transcriptional activation domains (AD1 and AD2) 42. AIB1 can interact with multiple transcription factors through different regions. To address whether AIB1 can interact with MafB to exert its co-activating function, we first performed co-immunoprecipitation assay using 293T cells transfected with Flag-tagged AIB1 and MafB expression vectors. As shown in Figure 5A, AIB1 interacted reciprocally with MafB. Then, we found that the AD2 domain of AIB1 was required for the interaction of AIB with MafB (Figure 5B). A GST pull-down assay was performed to confirm that AIB1 can directly bind to MafB through the AD2 domain (Figure 5C). In addition, the results showed that knocking down MafB also reduced the expression of SMO and ITGAV in PANC-1 cells (Figure S10D). Therefore, these results indicate that AIB1 is a newly discovered coactivator for MafB that promotes the transcription of target genes such as *SMO* and *ITGAV*.

### PDAC cells with high AIB1 levels are sensitive to blockage of Hh activation

**Figure 6:**
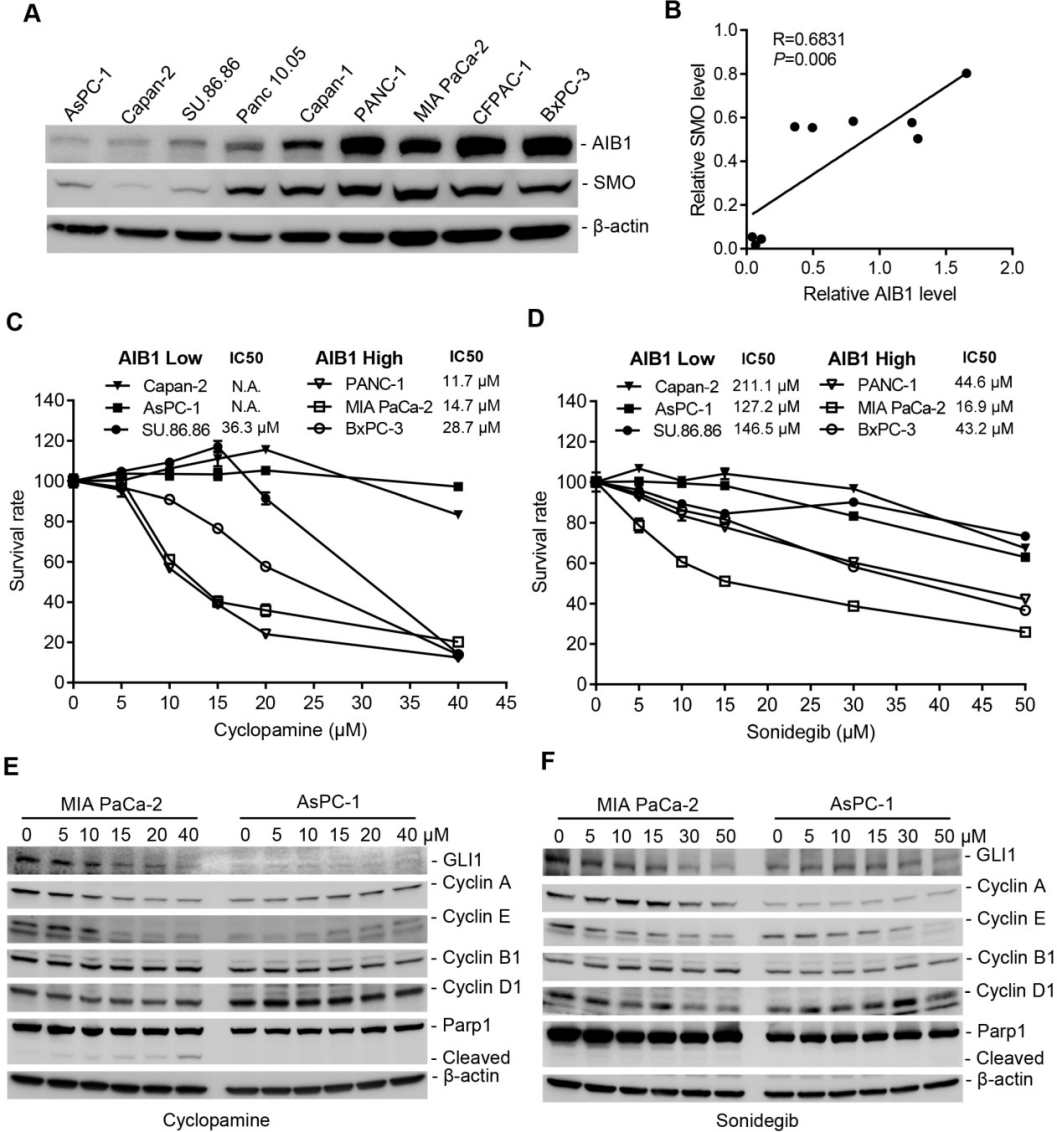
** Blockage of Hh signaling activation is effective for treating PDAC cells with high AIB1 levels.** (A, B) The levels of AIB1 have a good correlation with the levels of SMO in different PDAC cells. (A) The expression of AIB1 and SMO was analyzed by Western blotting; (B) The relationship between AIB1 levels and SMO levels. (C, D) The sensitivity of PDAC cells expressing high and low levels of AIB1 to SMO inhibitors, cyclopamine (C) and sonidegib (D). Data are presented as the means ± SEM. (E, F) SMO inhibitors decrease downstream targets of Hh signaling in MIA PaCa-2 cells with high AIB1 levels, (E) cyclopamine and (F) sonidegib.

The Hh pathway is aberrantly activated in a variety of human cancers, including PDAC. Therefore, most efforts have been directed to pharmacologically blocking the activity of the Hh pathway 43. We found that AIB1 levels showed a good correlation with SMO levels *in vitro* and *in vivo* (Figure 6A-B and Figure S6C), suggesting that PDAC cells with high AIB1 levels may be sensitive to Hh inhibition. In addition, the results showed that the level of SMO is also associated with the progression of PDAC (Figure S12). Therefore, we performed sensitivity test using SMO inhibitors treatment of three PDAC cell lines with high AIB1 expression (PANC-1, MIA PaCa-2 and BxPC-3) and three with low AIB1 expression (Capan-2, AsPC-1 and SU 86.86). The results indicated that the PDAC cell lines with AIB1 high expression were more sensitive to SMO inhibitors such as cyclopamine, sonidegib and GANT1 (Figure 6C-D and Figure S13A). Then, we investigated whether the effect of SMO inhibitors on PDAC cell with highly expressed AIB1 depends on Hh activation. The results showed that downstream targets of the Hh signaling pathway, such as GLI1, Cyclin A and E, were expressed at higher levels in MIA PaCa-2 cells than in AsPC-1 cells, and their expression was significantly reduced upon SMO inhibitor treatment (Figure 6E-F). In addition, we found PDAC cells with high AIB1 expression were also sensitive to GANT61, a GLI inhibitor, and they have a higher apoptotic rate than their counterparts with low AIB1 expression (Figure S13B-C). These results suggested that blocking Hh activation can effectively treat PDAC with high expression of AIB1.

**Figure 7:**
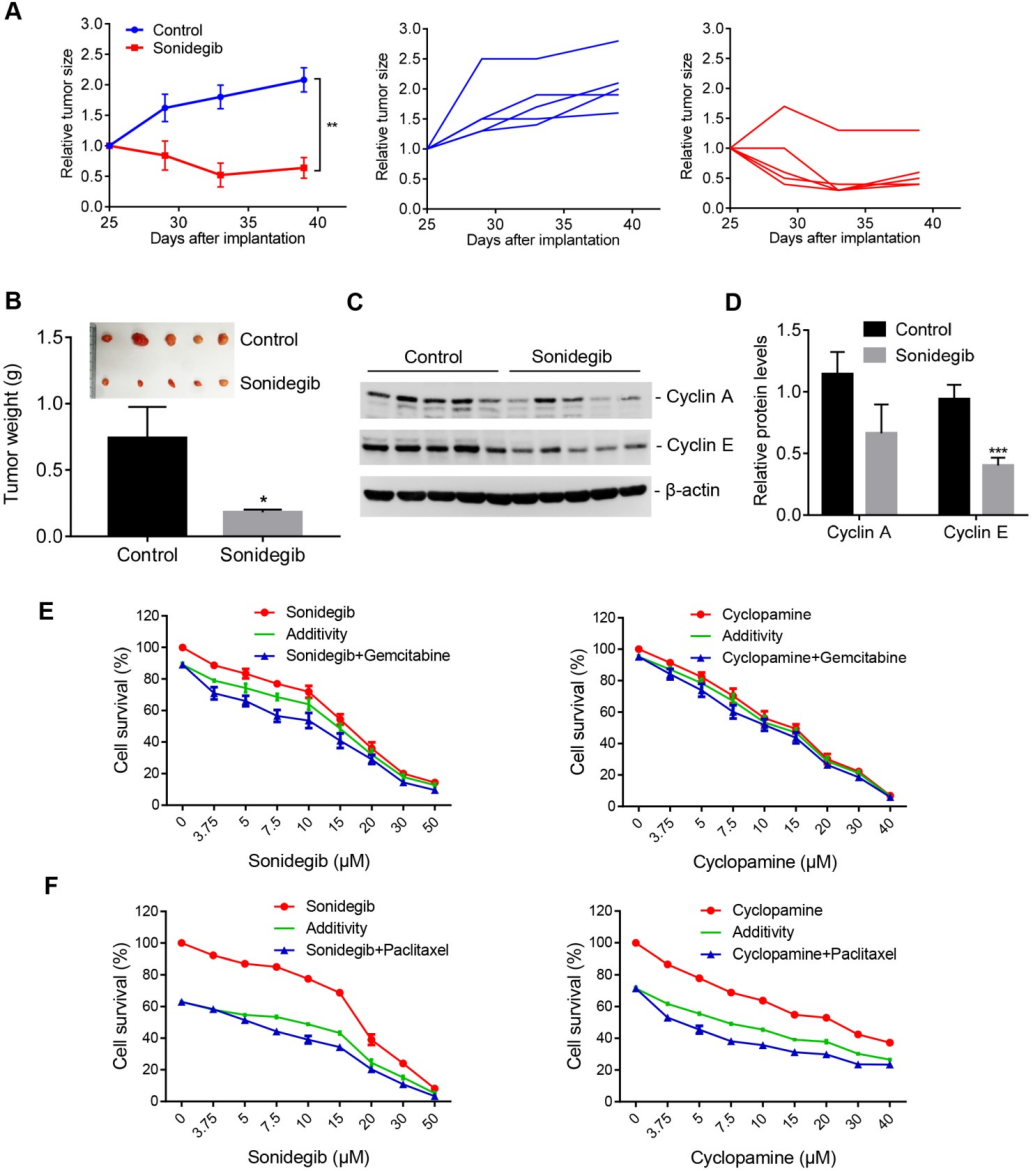
** SMO inhibitors have potential therapeutic effects on AIB1-high PDAC tumors.** (A-D) SMO inhibition suppressed the growth of MIA PaCa-2 cells *in vivo*. Mice were randomly assigned to two groups (n = 5 per group) and then treated with either vehicle (Control) or sonidegib. The effects of the drug on tumor size are shown by the growth curve (A), the excised tumors and tumor weight (B); the effects on cyclin A and E levels are shown in (C-D). (E, F) SMO inhibitors synergistically suppressed the proliferation of MIA PaCa-2 cells after gemcitabine (10 nM, E) or paclitaxel treatment (4 nM, F). Data are presented as the means ± SEM. *P < 0.05, **P < 0.01, ***P < 0.001 as indicated by two-tailed *t*-test (A, B and D).

To verify the efficacy of the SMO inhibitor on PDAC cells with high AIB1 expression *in vivo*, we inoculated MIA PaCa-2 and AsPC-1 cells into SCID mice, respectively. The results showed that sonidegib treatment significantly reduced the growth of MIA PaCa-2 tumors but not AsPC-1 tumors (Figure 7A-B and Figure S14A-B). The targets of Hh signalling, such as Cylin A and E, were also reduced in MIA PaCa-2 tumors after sonidegib treatment but not in AsPC-1 tumors (Figure 7C-D and Figure S14C-D). Gemcitabine and nab-paclitaxel are implemented as the standard of care for the first-line treatment of advanced pancreatic cancers. Therefore, we examined whether the SMO inhibitor has a synergistic effect on PDAC cells with high AIB1 expression treated with the standard of care. The results indicated that blocking Hh activation by sonidegib or cyclopamine synergistically inhibited the proliferation of PDAC cells with high AIB1 expression in combination with gemcitabine or paclitaxel treatment (Figure 7E-F).

**Figure 8:**
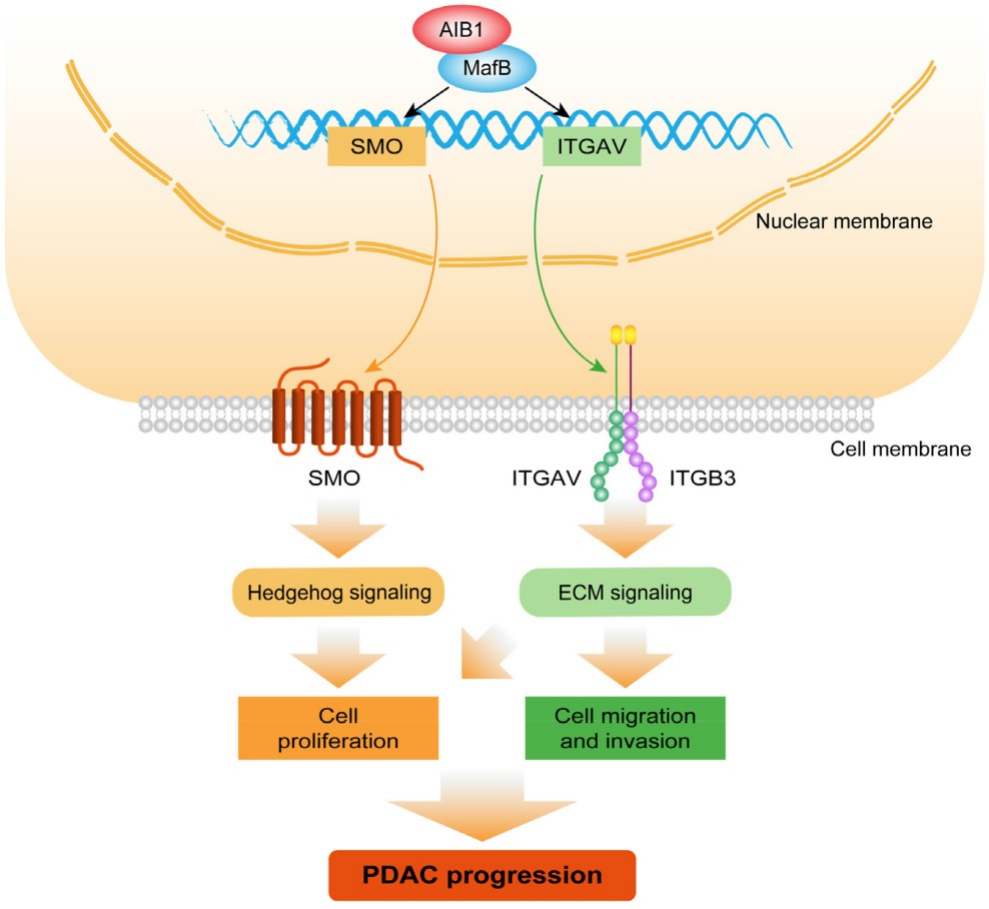
** Schematic model illustrating the role of AIB1 in PDAC progression.** AIB1 promotes the progression of PDAC through simultaneous activation of Hh and ECM signaling pathways.

Collectively, the findings of this study demonstrated that AIB1 promotes the proliferation and migration of PDAC cells *in vitro* and *in vivo* and that AIB1 interacts with MafB to promote the expression of SMO and ITGAV, which play important roles in hedgehog and ECM signaling, respectively (Figure 8). Furthermore, this study indicates that PDAC cells with high AIB1 levels are more sensitive to hedgehog inhibitors, which provides a novel potential strategy for precisely treating PDAC.

## Discussion

PDAC remains an aggressive cancer with poor prognosis and low survival. A recent study indicated that AIB1 is required for the tumor growth of PDAC and is considered as a potential target for this deadly disease 44. However, the underlying molecular mechanism remains unclear. In this study, we demonstrated that AIB1 promotes the progression of PDAC through simultaneous regulation of the Hh and ECM signaling pathways. AIB1 belongs to the steroid receptor coactivator family that interacts with nuclear receptors and with certain other transcription factors to mediate their activities. AP-1 is a dimeric transcription factor composed of DNA-binding proteins belonging to Jun, Fos, ATF and Maf subfamilies 45. Our previous study reported that AIB1 mediates the activity of JunB to increase matrix metallopeptidase-9 levels in HCC 15. Here, an analysis of global AIB1-binding motifs by ChIP-Seq indicated that AIB1 is significantly associated with AP-1 DNA-binding sites, including these four subfamilies, which suggests that AIB1 tends to interact with AP-1 associated proteins to regulate downstream targets. Our results reveal that AIB1 directly interacts with MafB, an AP-1 protein, *via* the AD2 domain, to promote the expression of SMO and ITGAV upstream of Hh and ECM signaling, respectively. The C-terminal domain of AIB1 (including AD1 and AD2) is its transcriptional activation domain, by which AIB1 interacts with HAT to facilitate the upregulation of gene expression by transcription factors, and this domain also has intrinsic HAT activity 16. These results imply that AIB1 is recruited to the regulatory regions of SMO and ITGAV through its interaction with MafB and upregulates histone acetylation to increase the accessibility of target genes.

Hh signaling, which has essential functions in developmental patterning, is frequently upregulated in PDAC 7. Hh activity is dependent upon the expression of SMO, an essential Hh response component that is frequently dysregulated in malignant tissues, including pancreatic cancerous tissues 46-50. Several groups have reported possible correlations between SMO expression and cancer prognosis, including in colorectal cancer and mesothelioma 48, 49. Although it has been reported that the expression of SMO is regulated by the level of promoter methylation 51, 52, the relevant molecular mechanism remains unclear. The transcription factor MafB is required for both α- and β-cell differentiation during pancreatic development, and then decreases in β cells and becomes a specific factor of adult islet α cells 53. Single-cell analysis has demonstrated that α cells are the most proliferative cells among endocrine cells and that Hh signaling is a candidate pathway for regulating α-cell proliferation 54, 55. This evidence suggests that MafB may regulate cell proliferation through Hh signaling. In this study, we found that MafB is critical for SMO expression and that AIB1 acts as a coactivator to enhance MafB-mediated transcriptional activation. In addition, the results indicate that AIB1 promotes PDAC cell proliferation, which is dependent on SMO. Overall, our study suggests that aberrant AIB1 expression in PDAC malignant tissues leads to Hh signaling dysregulation by mediating MafB activity, thereby promoting the progression of PDAC. However, it is not clear whether AIB1 plays an important role as a mediator of pancreatic carcinogenesis, and we will further address this possibility in future studies.

Dysregulated ECM signaling promotes malignant transformation and metastasis by fostering integrin-dependent cell adhesion and migration 11. FAK, a tyrosine kinase, is activated by both integrins and growth factors to increase focal adhesion turnover and promote cell migration 56. AIB1 has been implicated in FAK activation and focal adhesion turnover 57. Previous study has showed that an AIB1 splice isoform with a deletion of exon 4 (SRC-3Δ4) acts as a signaling adaptor, bridging the interaction of EGFR with FAK and promoting EGF-induced phosphorylation of FAK 58. However, it is unclear whether AIB1 is also involved in integrin-mediated FAK activation. Here, we found that AIB1 facilitates FAK activation and focal adhesion formation upon vitronectin stimulation by promoting the expression of ITGAV, a subunit of integrin αvβ3. ECM remodeling and changes in cellular interactions with ECM play crucial roles in the initiation and progression of the EMT 59. Our findings indicate that ECM signaling is required for the expression of the EMT activator ZEB1 in PDAC cells, but the relevant mechanism needs to be addressed in future studies. In addition, surprisingly, knocking down ITGAV reduced the level of its partner, ITGB3, at the protein level, which suggests that the formation of Integrin α_v_β_3_ may increase ITGB3 stability. Future studies should examine the underlying mechanism of integrin αVβ3 heterodimer coordination.

Aberrant Hh signaling occurs in the initiation and progression of PDAC 6, 9; hence, therapeutics that target Hh signaling may improve the outcomes of patients with PDAC. A variety of small molecules targeting SMO or GLI1 have been developed as strategies to inhibit Hh signaling 8. SMO inhibitors have shown high efficacy in patients with basal cell carcinoma (BCC) harboring activating mutations in the Hh pathway, leading to the approval of vismodegib and sonidegib, two SMO inhibitors, as therapies for advanced BCC 60. Therefore, a larger number of clinical trials in multiple cancers have explored whether the applicability of SMO inhibitors can be extended to cancers other than BCC 8, 61. However, the outlook is not as optimistic as previously thought, and negative results have been reported for many types of cancers, including PDAC 8. Notably, all of these negative trials were performed in unselected patients, suggesting that correlative biomarkers for predicting the efficacy of Hh inhibitors need to be developed for treatment. Our data indicate that AIB1 levels affects the sensitivity of PDAC cells to SMO inhibitors, implying that the inhibition of Hh signaling may be a strategy for treating patients with PDAC characterized by highly expressed AIB1.

## Conclusions

In summary, our study reveals that the nuclear coactivator AIB1 plays a critical role in facilitating PDAC progression *via* simultaneous enhancement of Hedgehog and ECM signaling. This finding raises an interesting possibility suggesting that suppression of AIB activity or that of its downstream targets, such as SMO and ITGAV, may be a good therapeutic strategy for PDAC.

## Supplementary Material

Supplementary figures and tables.

## Abbreviations

AD: activation domain
AIB1: amplified in breast cancer 1
BCC: Basal cell carcinoma
DEGs: Genes differentially expressed
ChIP: Chromatin immunoprecipitation
ECM: Extracellular matrix
EMT: Epithelial-mesenchymal transition
FAK: Focal adhesion kinase
FACS: Fluorescence-activated cell sorting
GO: Gene ontology
GSEA: Gene set enrichment analysis
HAT: Histone acetyltransferase
HCC: Hepatocellular carcinoma
Hh: Hedgehog
IP: Immunoprecipitation
i.p.: Intraperitoneally
ITGAV: Integrin αv
ITGB3: Integrin β3
KD: Knockdown
KEGG: Kyoto Encyclopedia of Genes and Genomes
MARE: MAF-recognition elements
PanIN: Pancreatic intraepithelial neoplasia
PTCH: Patched
bHLH/PAS: PAS (Per/ARNT/Sim) motif
PDAC: Pancreatic ductal adenocarcinoma
RNA-Seq: RNA sequencing
RID: Receptor-interaction domain
RTCA: Real time cell analyzer system
SCID: Severe combined immunodeficient
SHH: Sonic hedgehog
SMO: Smoothened
SRC-3: Steroid receptor coactivator-3
S/T: Serine/threonine-rich region
TCGA: The Cancer Genome Atlas
TF: Transcription factors
ZEB1: Zinc‐finger E‐box binding homeobox 1
