# Supplementary material for: Upregulation of amplified in breast cancer 1 contributes to pancreatic ductal adenocarcinoma progression and vulnerability to blockage of hedgehog activation
Source: Theranostics. 2021 Jan 1;11(4):1672–89. doi: 10.7150/thno.47390 (PMC7778610; doi:10.7150/thno.47390)
Supplement: Supplementary file 1 — Supplementary figures and tables. [file thnov11p1672s1.pdf]

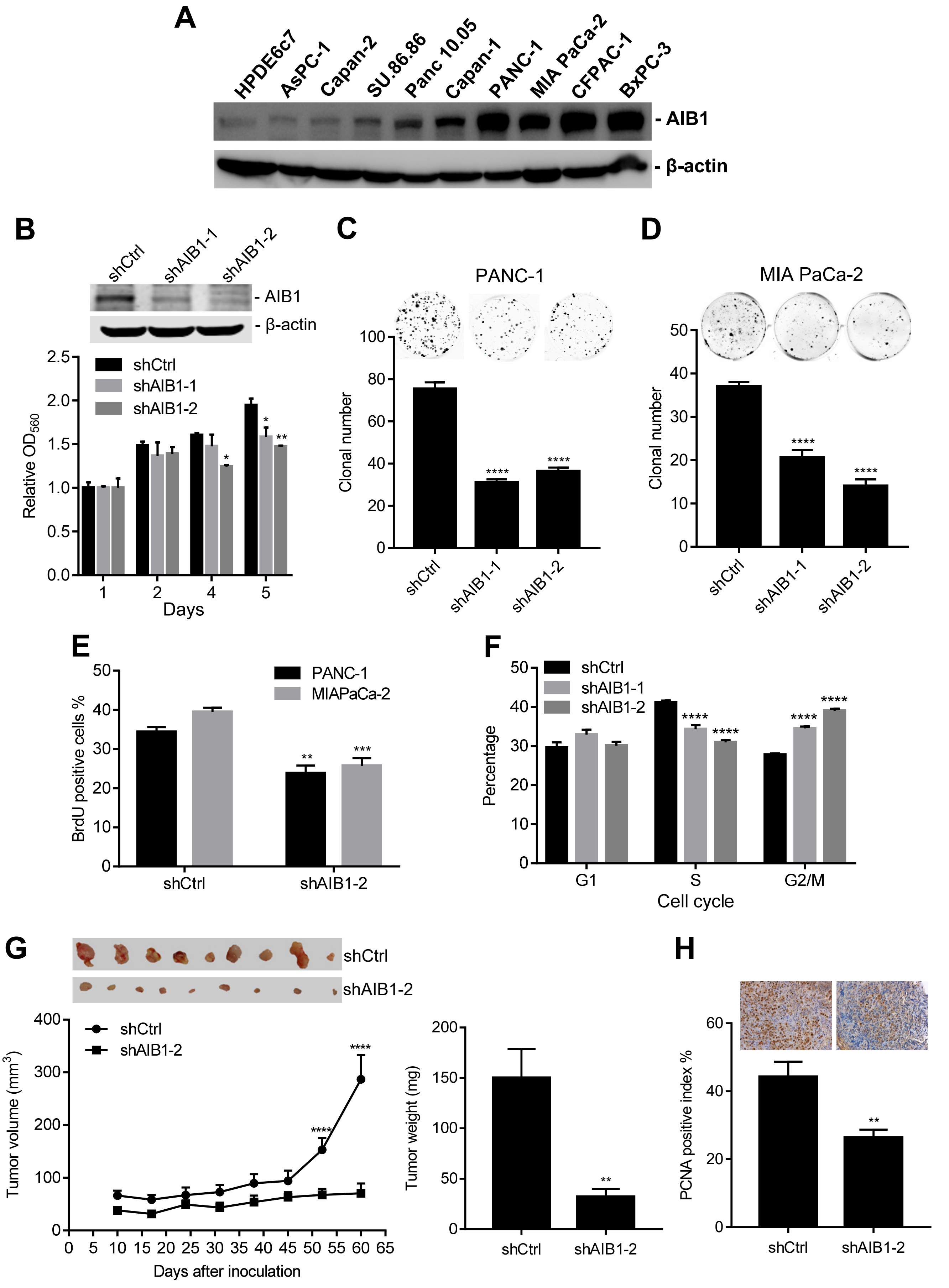
A
HPDE6c7
AsPC-1
Capan-2
SU.86.86
Panc 10.05
Capan-1
PANC-1
MIA PaCa-2
CFPAC-1
BxPC-3
- AIB1
- β-actin
B
shCtrl
shAIB1-1
shAIB1-2
- AIB1
- β-actin
Relative OD560
Days
C
PANC-1
Clonal number
D
MIA PaCa-2
Clonal number
E
BrdU positive cells %
PANC-1
MIAPaCa-2
F
Percentage
G1
S
G2/M
Cell cycle
G
shCtrl
shAIB1-2
Tumor volume (mm3)
Days after inoculation
Tumor weight (mg)
H
PCNA positive index %

Figure S1. Down-regulation of AIB1 inhibits proliferation of PDAC cells *in vitro* and *in vivo*. (A) The AIB1 level in different PDAC cell lines and HPDE6c7 cells determined by Western blotting. (B) The effect of AIB1 KD on proliferation of MIA PaCa-2 cells. Upper panel: Western blot for AIB1; lower panel: quantification of cell proliferation by MTT assay. (C and D) The oncogenic potential of AIB1 in PANC-1 (C) or MIA PaCa-2 (D) cells determined by colony formation assay. Upper panel: representative image of colonies; lower panel: quantification for colony formation. (E) BrdU incorporation of PANC-1 and MIA PaCa-2 cells. (F) Cell cycle analysis of PANC-1 cells with shCtrl or shAIB1. (G) AIB1 KD with shAIB1-2 decreased tumor growth of PANC-1 cells *in vivo*. n=9 per group. Upper left panel: the image of tumors; lower left panel: the curve of tumor growth; right panel: quantitative analysis of tumor weight. (H) Immunohistochemical analysis of PCNA levels in tumors from (G). Data represent the mean ± SEM. *$P < 0.05$, **$P < 0.01$, ***$P < 0.001$, ****$P < 0.0001$ by one-way (C and D)/two-way (B, E, and F) ANOVA or an unpaired two-tailed t test (G and H).

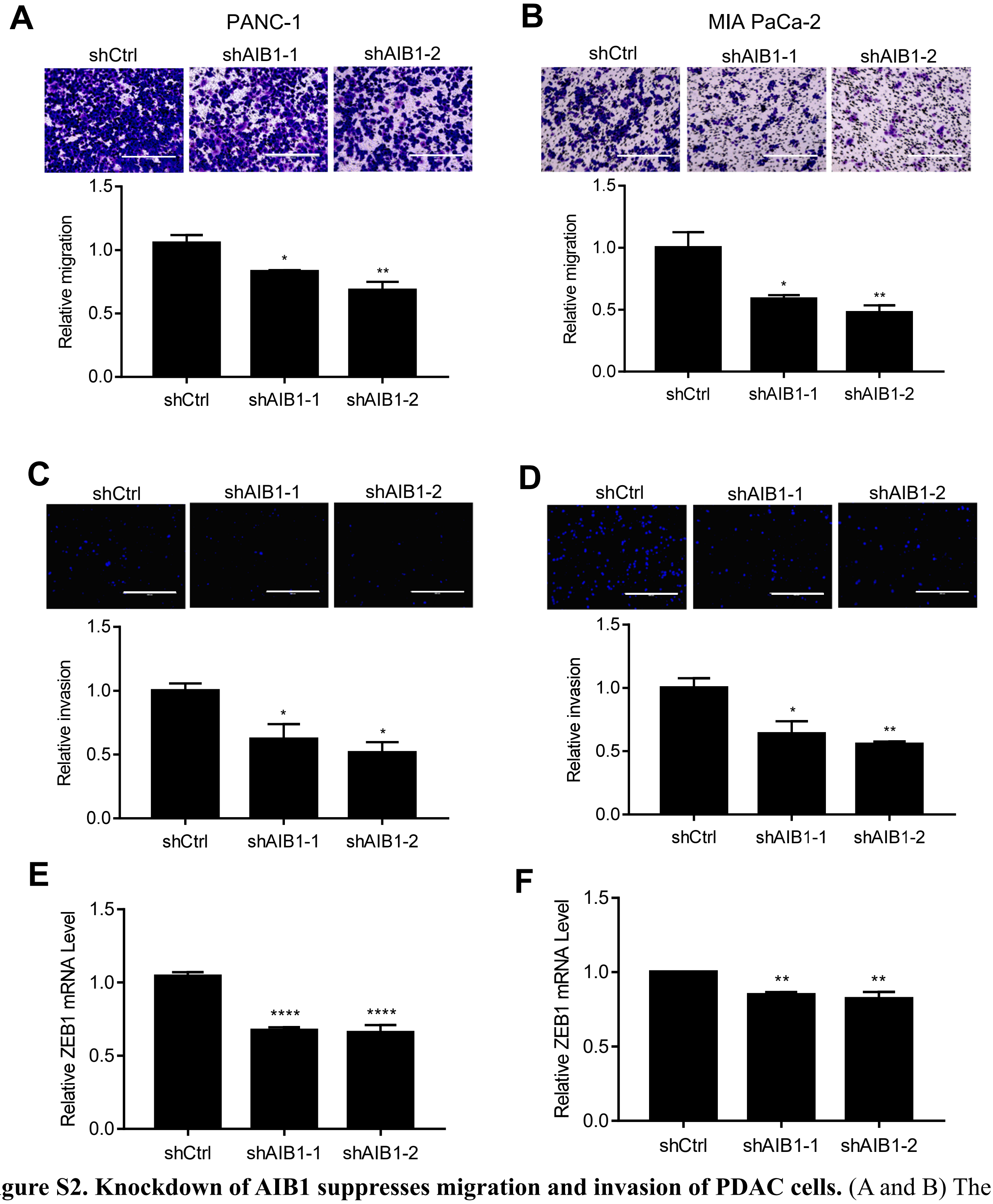

Figure S2. Knockdown of AIB1 suppresses migration and invasion of PDAC cells. (A and B) The effect of AIB1 on migration of PANC-1 (A) and MIA PaCa-2 (B) cells. Upper panel: representative images of transwell cell migration assay; lower panel: quantification of cell migration. Scale bar, 400 μm. (C and D) The effect of AIB1 on invasion of PANC-1 (C) and MIA PaCa-2 (D) cells. Upper panel: representative images of transwell cell invasion assay, lower panel: quantification of cell invasion. Scale bar, 400 μm. (E and F) The effect of AIB1 on the level of ZEB1 mRNA in PANC-1 (E) and MIA PaCa-2 (F) cells. Data represent the mean ± SEM. **P* < 0.05, ***P* < 0.01, *****P* < 0.0001 by one-way ANOVA.

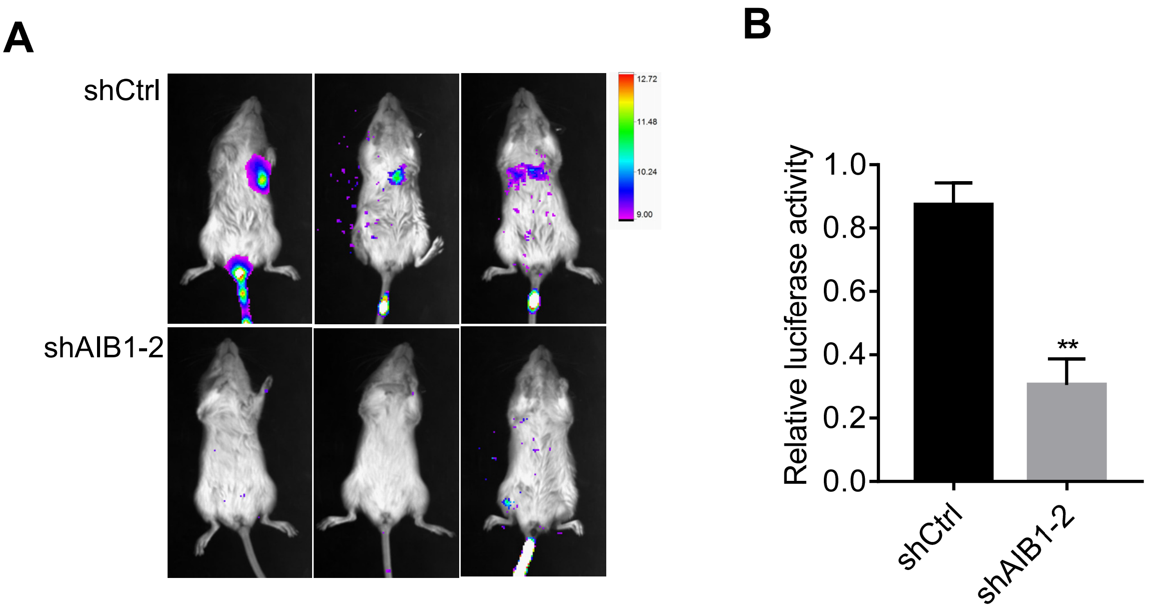

Figure S3. Knockdown of AIB1 suppresses the colonization of PDAC cells in the lung. shAIB1-2 and shCtrl Luc-GFP cells were intravenously injected into the mice through tail vein. After injection for 36 h, the luciferase activity in the lung was detected. (A) Representative images of mice. (B) Quantitation for luciferase activity in the lungs, n=3 per group. Data represent the mean ± SEM. ***P* < 0.01 by an unpaired two-tailed t test

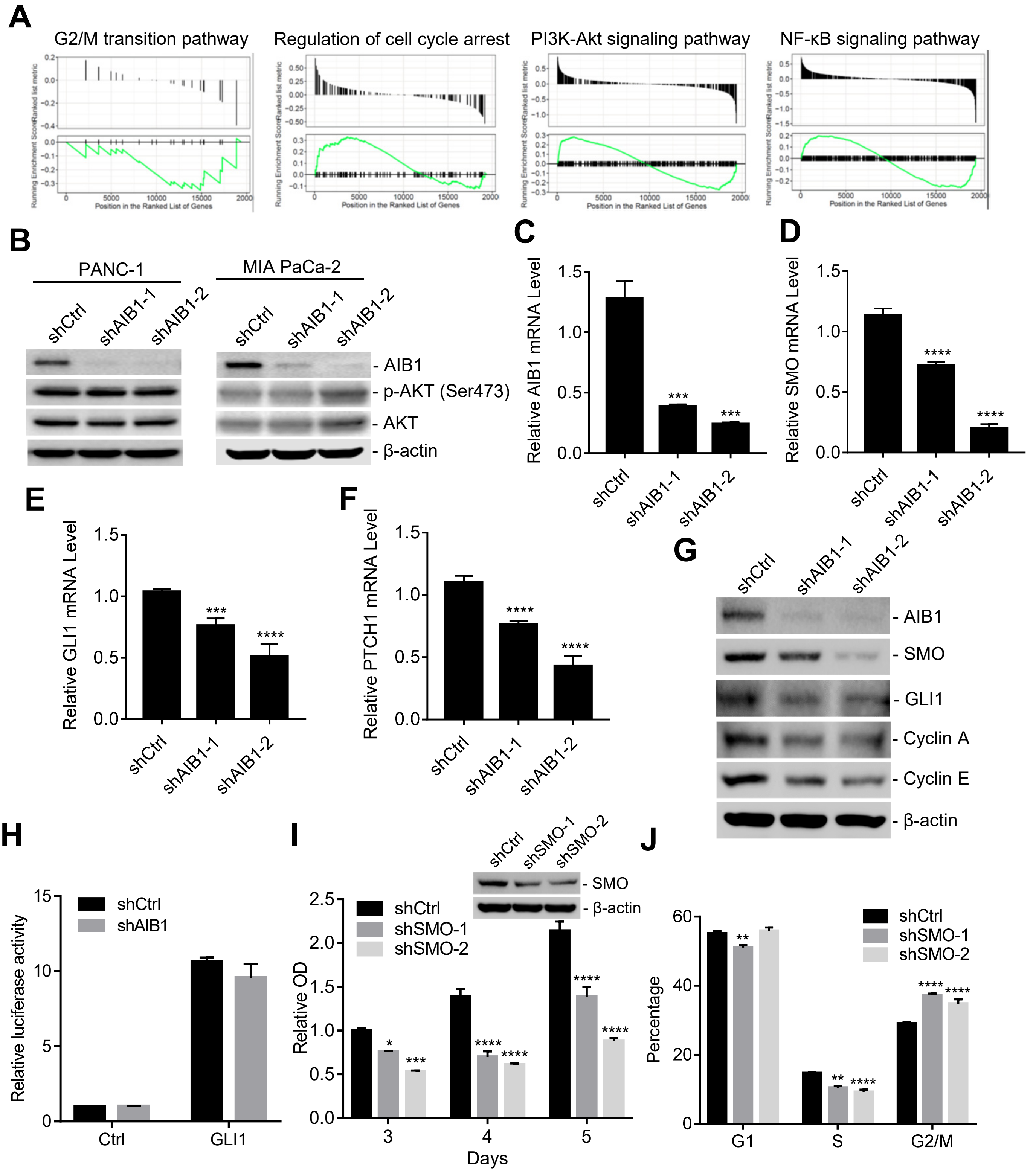

Figure S4. AIB1 regulates the proliferation of PDAC cells dependent of Hh signaling, not AKT or NF-κB activation. (A) GSEA plot of enrichment in “G2/M transition pathway”, “Regulation of cell cycle arrest”, “PI3K-Akt signaling pathway” and “NF-κB signaling pathway” gene sets. (B) AIB1 has no effect on AKT phosphorylation in PDAC cells. (C-F) The effect of AIB1 on Hh signaling in PANC-1 cells by real-time PCR. (G) The effect of AIB1 on the expression of SMO, GLI1, Cyclin A and Cyclin E in MIA PaCa-2 cells. (H) AIB1 had no effect on Hh activation induced by overexpression of GLI1. (I and J) Downregulation of SMO suppressed the proliferation (I) and caused cell cycle arrest (J) in MIA PaCa-2 cells . Data represent the mean ± SEM. * P<0.05, ** $P < 0.01$, *** $P < 0.001$, **** $P < 0.0001$ by ANOVA.

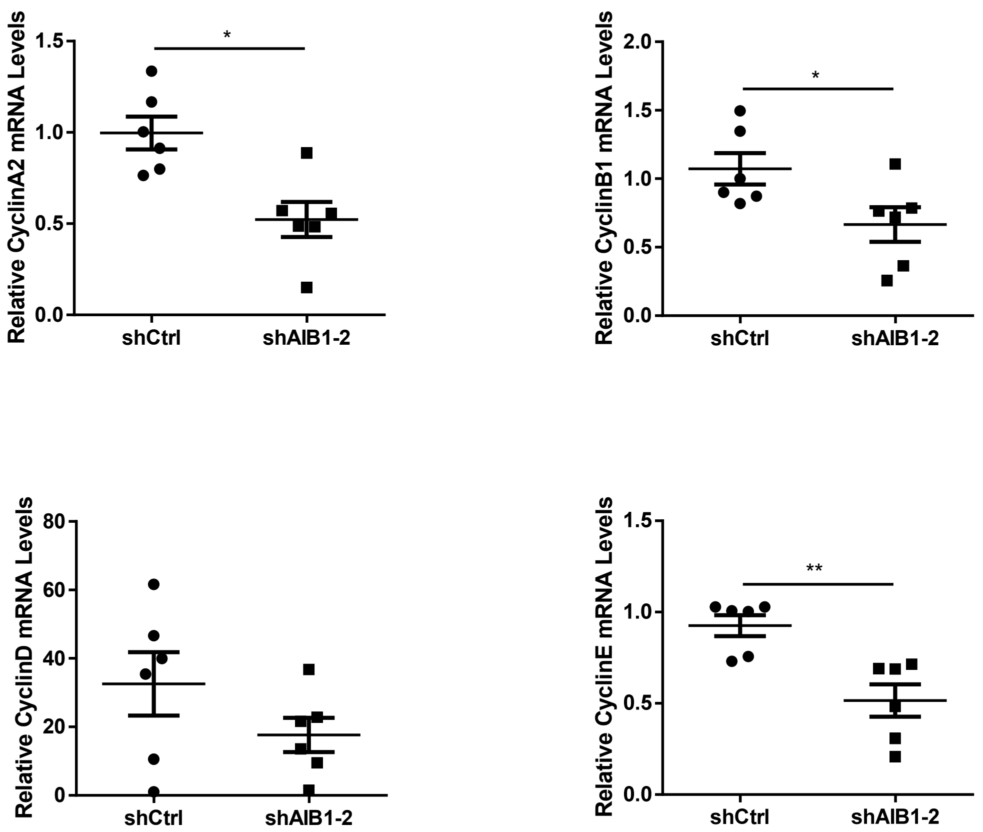

Figure S5. The effect of AIB1 on the expression of Cyclins in xenograft tumors. n=6 per group. Data represent the mean ± SEM. * P<0.05, **P < 0.01 by an unpaired two-tailed t test

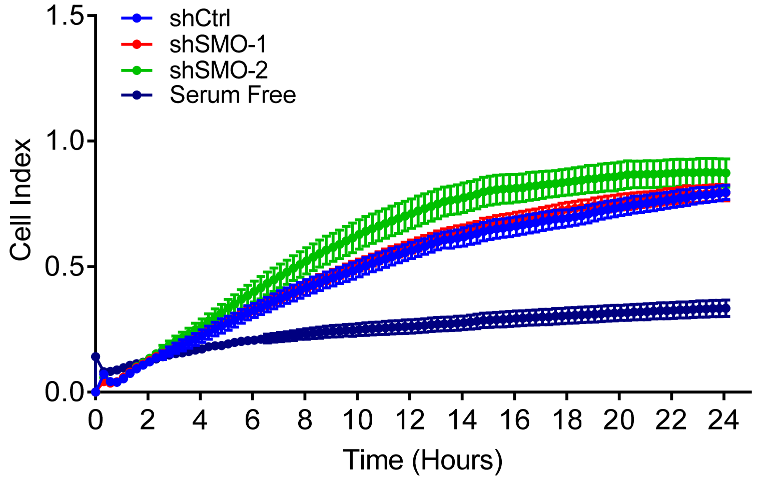

Figure S6. Downregulation of SMO has no effect on migration of PANC-1 cells.

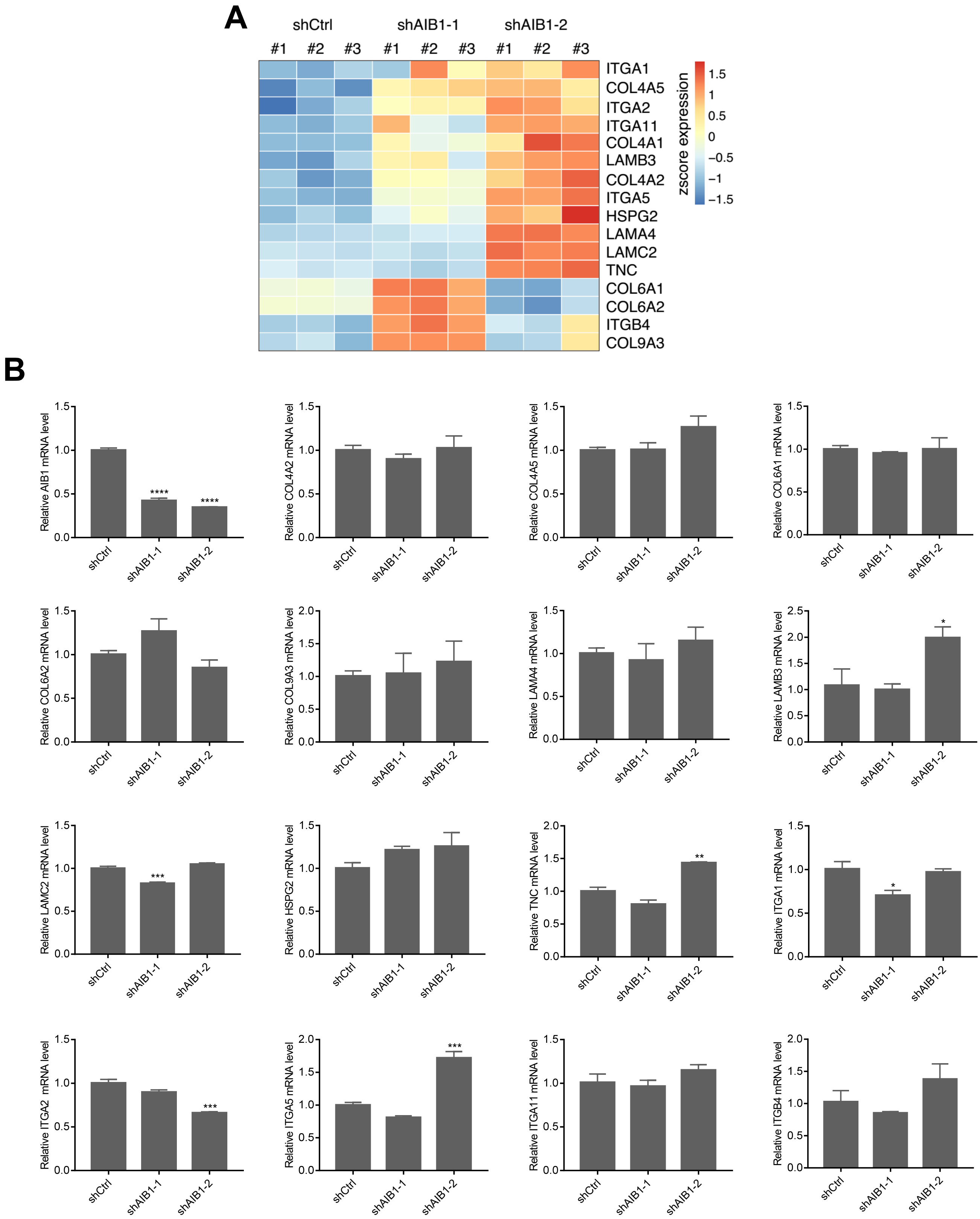

Figure S7. Up-regulation of ECM-receptor interaction pathway in AIB1 stable KD cells is the secondary event. (A) The heatmap of 16 genes related to ECM-receptor interaction pathway. (B) The mRNA levels of these genes in PANC-1 cells after transient KD of AIB1. Data represent the mean ± SEM. $*P < 0.05$, $**P < 0.01$, $***P < 0.001$, $****P < 0.0001$ by one-way ANOVA.

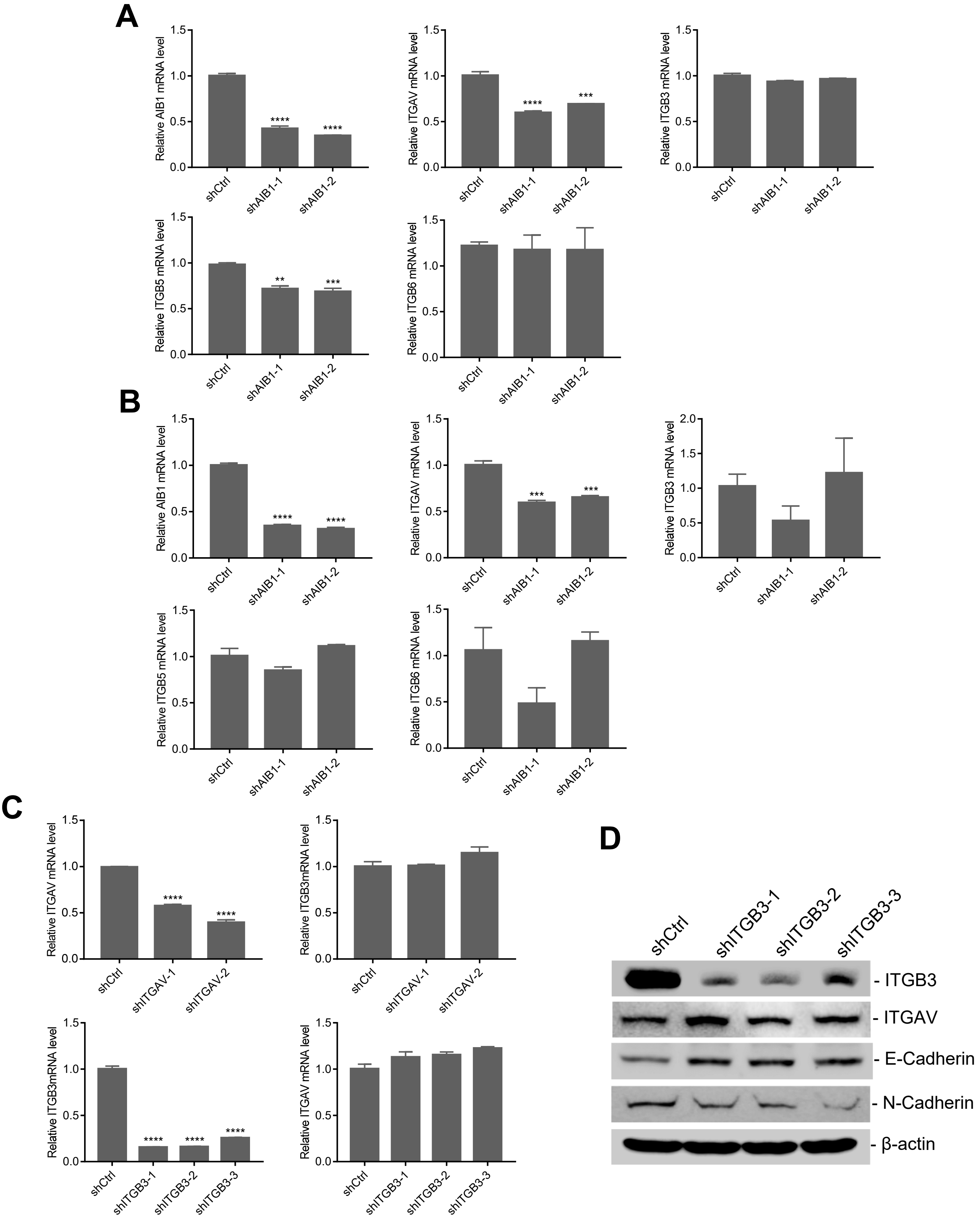

Figure S8. Transient KD of AIB1 inhibits the expression of ITGAV. (A, B) The effect of AIB1 on the expression of integrins in PANC-1 (A) and MIA PaCa-2 (B) cells. (C) ITGAV and ITGB3 have no effect on each other at the mRNA level. (D) ITGB3 KD induced cadherin switching but had no effect on the expression of ITGAV in PANC-1 cells. Data represent the mean ± SEM. ***P* < 0.01, ****P* < 0.001, *****P* < 0.0001 by one-way ANOVA.

A

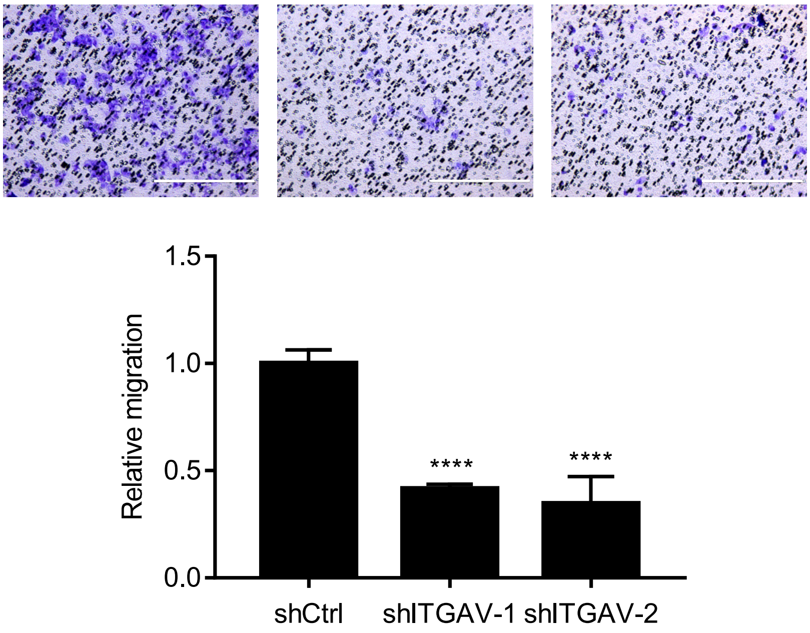

B

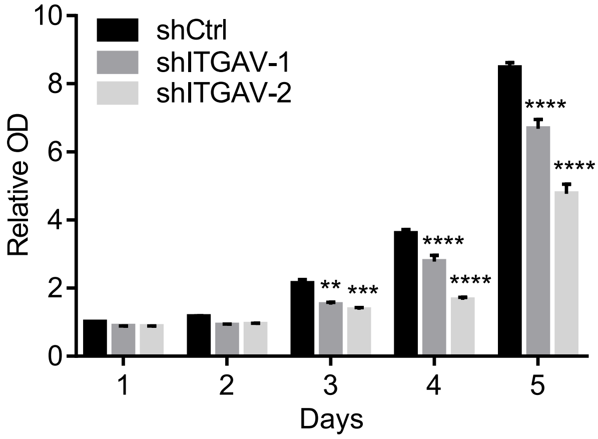

Figure S9. The effect of ITGAV on the migration and proliferation of MIA PaCa-2 cells. (A) Migration. Scale bar, 400 μm. (B) Proliferation. Data represent the mean ± SEM. $^{**}P < 0.01$, $^{***}P < 0.001$, $^{****}P < 0.0001$ by one-way ANOVA.

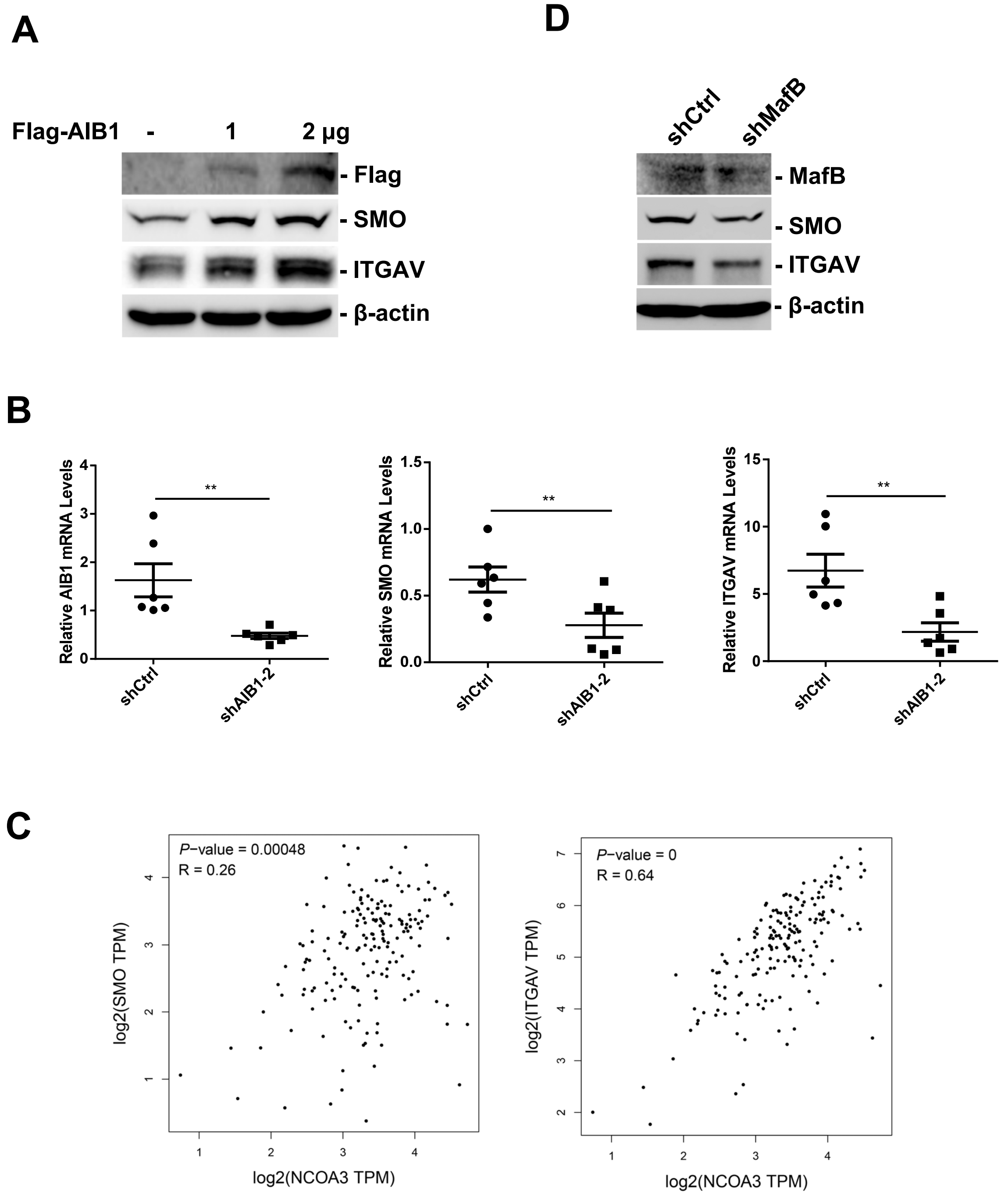

Figure S10. AIB1 regulates the expression of SMO and ITGAV via mediating transcriptional activity of MafB. (A) Overexpression of AIB1 increased the levels of SMO and ITGAV in Capan-2 cells. (B) The expression SMO and ITGAV in xenograft tumors with shAIB1-2. n=6 per group. (C) The correlation of AIB1 levels with SMO levels or ITGAV levels in clinical PDAC samples which were extracted from TCGA database by GEPIA, n=179. (D) MafB KD decreased the expression of SMO and ITGAV in PANC-1 cells. Data represent the mean ± SEM. ***P* < 0.01 by unpaired two-tailed t test.

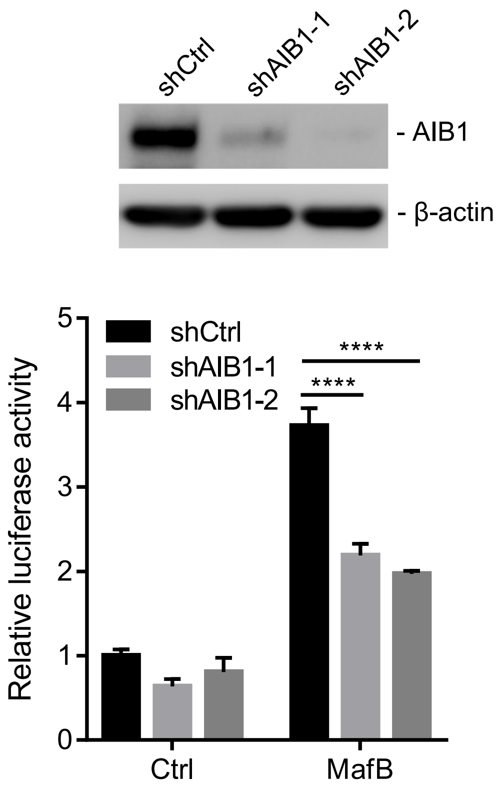

Figure S11. AIB1 KD impairs MafB-mediated SMO promoter activity in MIA PaCa-2 cells. Data represent the mean ± SEM. ****$P < 0.0001$ by two-way ANOVA.

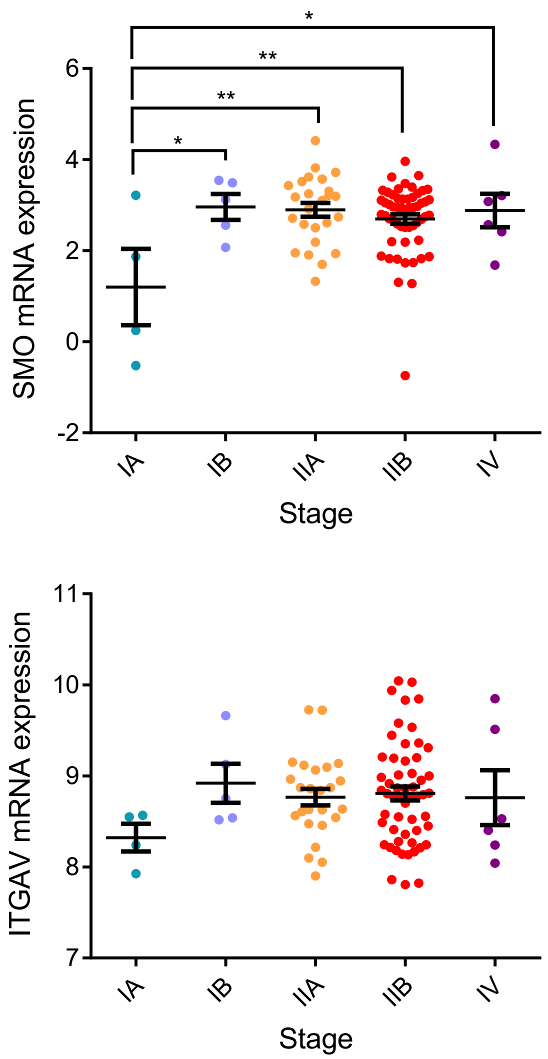

Figure S12. The SMO and ITGAV mRNA levels in different stages of PDAC. *$P < 0.05$, ** $P < 0.01$ by one-way ANOVA.

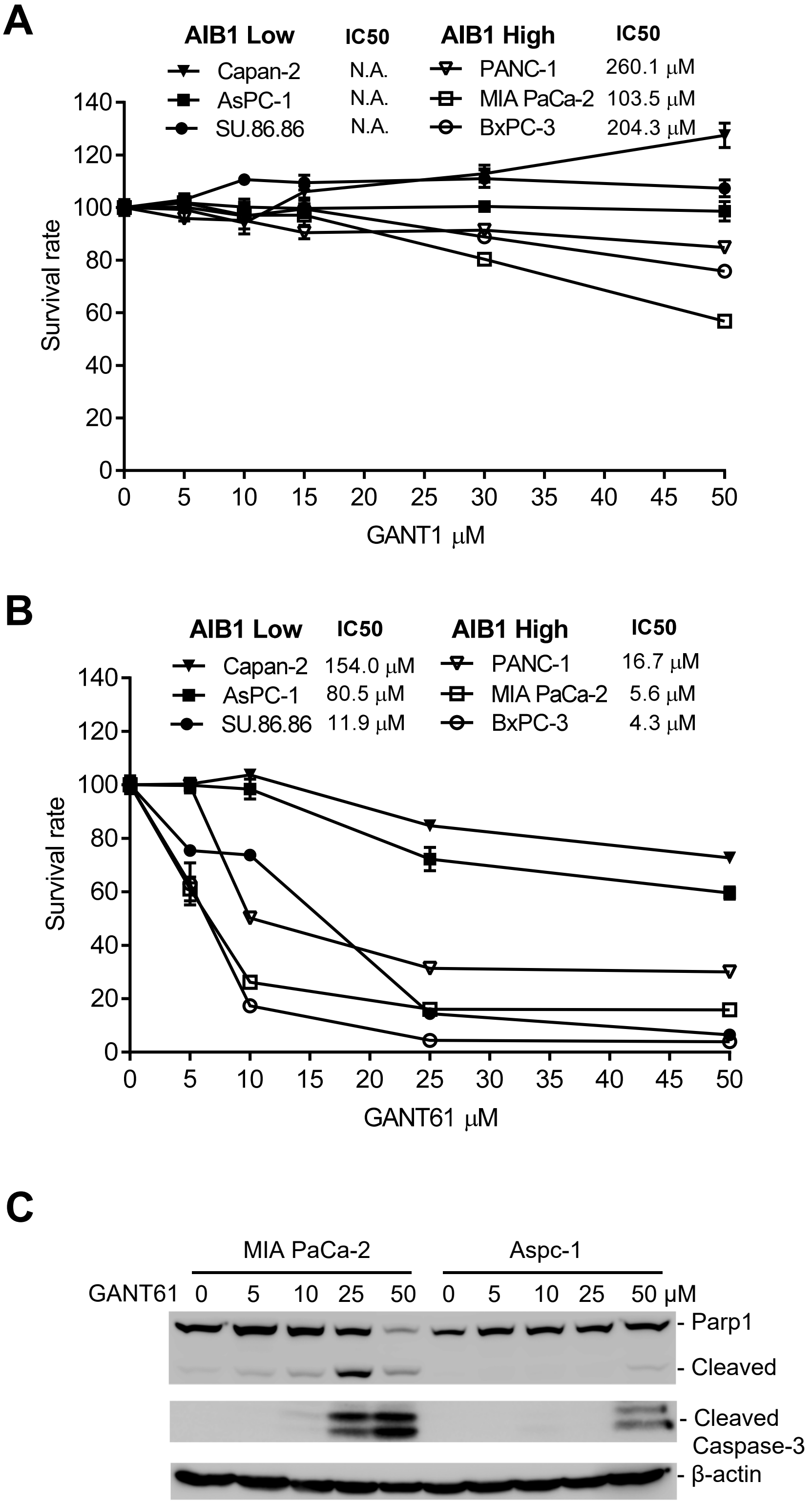

Figure S13. PDAC cells with high AIB1 level are sensitive to blockage of Hh signaling activation. (A and B) The sensitivity of AIB1 high and low PDAC cells to GANT1 (A) and GANT61 (B). Data are represented as mean ± SEM. (C) MIAPaCa-2 cells with high AIB1 level had more apoptosis under GANT61 treatment than Aspc-1 cells with low AIB1 level.

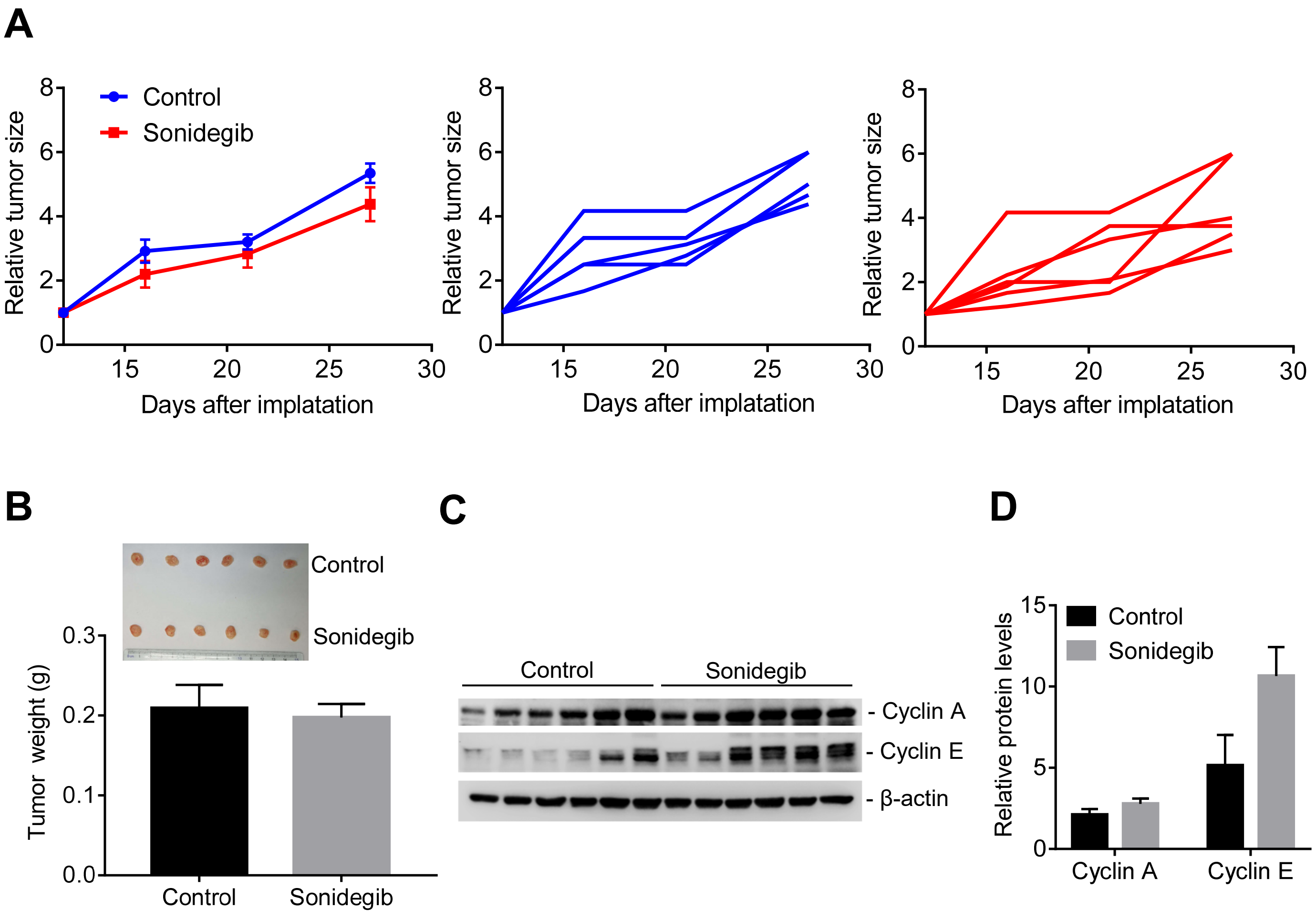

Figure S14. PDAC cell with low AIB1 level is resistant to SMO inhibitor. SMO inhibition has no effect on the growth of AsPC-1 *in vivo*. Mice were randomized into two groups (n = 6 per group) and then treated with either vehicle (Control) or Sonidegib. The effects of drug on tumor size are shown by growth curve (A), the excised tumors and tumor weight (B), and the effects on cyclin A and E levels are shown in (C-D).

Table S1. Primers used for experiments

Target	Species	sequence 5'-3'
**AIB1 F**	Human	TTCCATCAAACCCTGAGAGC
**AIB1 R**	Human	GACGTTTCTGGGACCATGAC
**SMO F**	Human	TCGAATCGCTACCCTGCTG
**SMO R**	Human	CAAGCCTCATGGTGCCATCT
**GLI1 F**	Human	AGCGTGAGCCTGAATCTGTG
**GLI1 R**	Human	CAGCATGTACTGGGCTTTGAA
**PTCH F**	Human	CCAGAAAGTATATGCACTGGCA
**PTCH R**	Human	GTGCTCGTACATTTGCTTGGG
**Cyclin A2 F**	Human	AGCTGCCTTTCATTTAGCACTCTAC
**Cyclin A2 R**	Human	TTAAGACTTTCCAGGGTATATCCAGTC
**Cyclin B1 F**	Human	TACCTATGCTGGTGCCAGTG
**Cyclin B1 R**	Human	CACATCCAGATGTTTCCATTG
**Cyclin D F**	Human	AGAGGCGGAGGAGAACAAAC
**Cyclin D R**	Human	TGAGGCGGTAGTAGGACAGG
**Cyclin E F**	Human	GCCAGCCTTGGGACAATAATG
**Cyclin E R**	Human	CTTGCACGTTGAGTTTGGGT
**COL4A2 F**	Human	TTATGCACTGCCTAAAGAGGAGC
**COL4A2 R**	Human	CCCTTAACTCCGTAGAAACCAAG
**COL4A5 F**	Human	CAAAAGGTGATCGTGGTTTCCC
**COL4A5 R**	Human	GTCCAGGTTGTCCATTTGGTC
**COL6A1 F**	Human	ACAGTGACGAGGTGGAGATCA
**COL6A1 R**	Human	GATAGCGCAGTCGGTGTAGG
**COL6A2 F**	Human	GACTCCACCGAGATCGACCA
**COL6A2 R**	Human	CTTGTAGCACTCTCCGTAGGC
**COL9A3 F**	Human	GTGGATGGTCTGACTGGACG
**COL9A3 R**	Human	GGGCAGATACTTGGGCACTG
**LAMA4 F**	Human	CCAGTGTAGGAATTGCTTACGC
**LAMA4 R**	Human	TAACCGCAGGTCATCAGTCAG
**LAMB3 F**	Human	CCAAAGGTGCGACTGCAATG
**LAMB3 R**	Human	AGTTCTTGCCTTCGGTGTGG
**LAMC2 F**	Human	TGGAGAACGCTGTGATAGGTG
**LAMC2 R**	Human	CAGGAGACCCATTTCGTTGGA
**HSPG2 F**	Human	GTGTGGTGTTCATCAAGGAGC
**HSPG2 R**	Human	GGGAGAGGTGACGTAGGAGG
**TNC F**	Human	TCCCAGTGTTCGGTGGATCT
**TNC R**	Human	TTGATGCGATGTGTGAAGACA
**ITGA1 F**	Human	CTGGACATAGTCATAGTGCTGGA
**ITGA1 R**	Human	ACCTGTGTCTGTTTAGGACCA

**ITGA2 F**	Human	AGGTGGGGTTAATTCAGTATGCC
**ITGA2 R**	Human	GATGTCTGGGATGTTGCTACAA
**ITGA5 F**	Human	GGCTTCAACTTAGACGCGGAG
**ITGA5 R**	Human	TGGCTGGTATTAGCCTTGGGT
**ITGA11 F**	Human	GTGGCAATAAGTGGCTGGTC
**ITGA11 R**	Human	GTTCCCGTGGATCACTGGAC
**ITGB4 F**	Human	GCTTCACACCTATTTCCCTGTC
**ITGB4 R**	Human	GACCCAGTCCTCGTCTTCTG
**ITGAV F**	Human	ATCTGTGAGGTCGAAACAGGA
**ITGAV R**	Human	TGGAGCATACTCAACAGTCTTTG
**ITGB3 F**	Human	CATGAAGGATGATCTGTGGAGC
**ITGB3 R**	Human	AATCCGCAGGTTACTGGTGAG
**ITGB5 F**	Human	AACTCGCGGAGGAGATGAG
**ITGB5 R**	Human	GGTGCCGTGTAGGAGAAAGG
**ITGB6 F**	Human	TCCATCTGGAGTTGGCGAAAG
**ITGB6 R**	Human	TCTGTCTGCCTACACTGAGAG
**18S F**	Human	AGTCCCTGCCCTTTGTACACA
**18S R**	Human	CGATCCGAGGGCCTCACTA
**SMO ChIP F**	Human	GCGAGCTAGAGCAACAAAGG
**SMO ChIP R**	Human	CATTCCCTCTCCCACCATTA
**ITGAV ChIP F**	Human	CATCTAGCCCAGACACAGGA
**ITGAV ChIP R**	Human	TGCCTGTTGCCTTAAGTTGA

Table S2 Antibodies used for experiments

WB/IP Antibody	Source	Identifier
AIB1	Cell Signaling Technology	Cat# 5765; RRID:AB_2797620
β-actin	Sigma	Cat# A5316; RRID:AB_476743
E-Cadherin	Cell Signaling Technology	Cat# 14472; RRID: AB_2728770
N-Cadherin	Abcam	Cat# ab76057; RRID: AB_1310478
Vimentin	Cell Signaling Technology	Cat# 5741; RRID: AB_10695459
ZEB1	Cell Signaling Technology	Cat# 3396; RRID: AB_1904164
SMO	Santa Cruz Biotechnology	Cat# sc-166685; RRID: AB_2239686
GLI1	Cell Signaling Technology	Cat# 2643; RRID: AB_2294746
Cyclin A	Santa Cruz Biotechnology	Cat# sc-751; RRID: AB_631329
Cyclin E	Santa Cruz Biotechnology	Cat# sc-481; RRID: AB_2275345
Cyclin B1	Santa Cruz Biotechnology	Cat# sc-245; RRID: AB_627338
Cyclin D1	Santa Cruz Biotechnology	Cat# sc-717; RRID: AB_631336
ITGAV	Cell Signaling Technology	Cat# 4711; RRID: AB_2128178
ITGB3	Cell Signaling Technology	Cat# 13166; RRID: AB_2798136
ITGB1	Cell Signaling Technology	Cat# 9699; RRID: AB_11178800
ITGB4	Cell Signaling Technology	Cat# 14803; RRID: AB_2798620
ITGB5	Cell Signaling Technology	Cat# 3629; RRID: AB_2249358
ITGA5	Cell Signaling Technology	Cat# 4705; RRID: AB_2233962
p-FAK Y397	Cell Signaling Technology	Cat# 8556; RRID: AB_10891442
p-FAK Y576/577	Cell Signaling Technology	Cat# 3281; RRID: AB_331079
p-FAK Y925	Cell Signaling Technology	Cat# 3284; RRID: AB_10831810
FAK	Cell Signaling Technology	Cat# 13009; RRID: AB_2798086
MafB	Santa Cruz Biotechnology	Cat# sc-376387; RRID: AB_10988929
Flag	Sigma	Cat# F1804; RRID: AB_262044
GST	Santa Cruz Biotechnology	Cat# sc-138; RRID: AB_627677
Parp1	Cell Signaling Technology	Cat# 9532; RRID: AB_659884
Cleaved Caspase-3	Cell Signaling Technology	Cat# 9664; RRID: AB_2070042
p-AKT S473	Cell Signaling Technology	Cat# 4060; RRID: AB_2315049
AKT	Cell Signaling Technology	Cat# 9272; RRID: AB_329827

ChIP Antibodies	Source	Catalog #
AIB1	Cell Signaling Technology	Cat# 2126; RRID: AB_823642
H3K27Ac	Abcam	Cat# ab4729; RRID: AB_2118291

IHC Antibodies	Source	Catalog #
PCNA	Abcam	Cat# ab18197; RRID: AB_444313
